# Carfentanil is a β‐arrestin‐biased agonist at the μ opioid receptor

**DOI:** 10.1111/bph.16084

**Published:** 2023-05-18

**Authors:** Nokomis Ramos‐Gonzalez, Sam Groom, Katy J. Sutcliffe, Sukhvinder Bancroft, Chris P. Bailey, Richard B. Sessions, Graeme Henderson, Eamonn Kelly

**Affiliations:** ^1^ School of Physiology, Pharmacology and Neuroscience University of Bristol Bristol UK; ^2^ Department of Pharmacy and Pharmacology University of Bath Bath UK; ^3^ School of Biochemistry University of Bristol Bristol UK

**Keywords:** bias, carfentanil, fentanyl, G protein, β‐arrestin

## Abstract

**Background and Purpose:**

The illicit use of fentanyl‐like drugs (fentanyls), which are μ opioid receptor agonists, and the many overdose deaths that result, has become a major problem. Fentanyls are very potent in vivo, leading to respiratory depression and death. However, the efficacy and possible signalling bias of different fentanyls is not clearly known. Here, we compared the relative efficacy and bias of a series of fentanyls.

**Experimental Approach:**

For agonist signalling bias and efficacy measurements, Bioluminescence Resonance Energy Transfer experiments were undertaken in HEK293T cells transiently transfected with μ opioid receptors, to assess Gi protein activation and β‐arrestin 2 recruitment. Agonist‐induced cell surface receptor loss was assessed using an enzyme‐linked immunosorbent assay, whilst agonist‐induced G protein‐coupled inwardly rectifying potassium channel current activation was measured electrophysiologically from rat locus coeruleus slices. Ligand poses in the μ opioid receptor were determined in silico using molecular dynamics simulations.

**Key Results:**

Relative to the reference ligand DAMGO, carfentanil was β‐arrestin‐biased, whereas fentanyl, sufentanil and alfentanil did not display bias. Carfentanil induced potent and extensive cell surface receptor loss, whilst the marked desensitisation of G protein‐coupled inwardly rectifying potassium channel currents in the continued presence of carfentanil in neurones was prevented by a GRK2/3 inhibitor. Molecular dynamics simulations suggested unique interactions of carfentanil with the orthosteric site of the receptor that could underlie the bias.

**Conclusions and Implications:**

Carfentanil is a β‐arrestin‐biased opioid drug at the μ receptor. It is uncertain how such bias influences in vivo effects of carfentanil relative to other fentanyls.

**Abbreviations**
aCSF: artificial cerebrospinal fluid
BRET: Bioluminescence Resonance Energy Transfer
BUDE: Bristol University Docking Engine
Cryo‐EM: cryogenic electron microscopy
DMEM: Dulbecco's modified Eagle's medium
ECL: extracellular loop
GAFF: general Amber force field
GIRK: G protein‐coupled inwardly rectifying potassium channel
GRK: G protein‐coupled receptor kinase
HA: human influenza hemagglutinin
HEK293T: human embryonic kidney 293
LC: locus coeruleus
MDs: molecular dynamics simulation
NA: noradrenaline
PDB: protein data bank
TBS: tris‐buffered saline
TM: transmembrane
β‐FNA: β‐funaltrexamine

**What is already known**

- “Fentanyls” such as fentanyl and carfentanil are potent agonists at the μ opioid receptor.

**What does this study add**

- Carfentanil is a β‐arrestin‐biased agonist at the μ opioid receptor whilst fentanyl is not biased.

**What is the clinical significance**

- Upon in vivo administration carfentanil may produce effects not seen with non‐biased fentanyls.

## 1 INTRODUCTION

### 1.1 The μ opioid receptor

The μ opioid receptor is a Gi/o G‐protein coupled receptor (GPCR) and agonist activation brings about potent analgesia as well as other effects including respiratory depression and euphoria through downstream signalling initiated largely by activation of G proteins (Matthes et al., 1996). The α subunit of the G protein inhibits adenylyl cyclase, whilst the Gβγ subunits, which remain tightly associated, activate inwardly rectifying potassium channels (GIRK) and inhibit voltage‐activated calcium channels (Pathan & Williams, 2012; Williams et al., 2013). Together, these actions serve to prevent further propagation of neuronal signalling (Al‐Hasani & Bruchas, 2011). GPCR kinases (GRKs) also phosphorylate the agonist‐bound μ opioid receptor allowing for the recruitment of β‐arrestins, leading to desensitisation of the G protein responses (Bailey et al., 2009; Kelly et al., 2008) and the internalisation of the receptor and potentially further signalling through the action of β‐arrestins as scaffold proteins (Shenoy & Lefkowitz, 2011).

### 1.2 Fentanyls

The synthetic μ opioid receptor agonist fentanyl is 50–100 times more potent than morphine in vivo (Hill et al., 2020), but overall has a similar effect profile to morphine, causing analgesia, sedation, respiratory depression, constipation and euphoria, as well as tolerance and dependence (Kelly, Sutcliffe, et al., 2023; Suzuki & El‐Haddad, 2017). Fentanyl was first synthesised in 1960 by Paul Janssen and is widely used in medicine as an adjunct to general anaesthetics and in the form of fentanyl patches (Peng & Sandler, 1999) to manage chronic and breakthrough pain (Stanley, 2014). Several analogues of fentanyl have also been developed for medical use, including sufentanil, alfentanil and remifentanil (Burns et al., 2018; Kissin, 2010). Carfentanil, an extremely potent analogue, is around 10,000 times more potent than morphine in vivo and was developed for use as a tranquiliser for large animals in veterinary medicine (Zawilska et al., 2021).

In recent years, there has been a marked rise in overdose deaths involving fentanyl and other synthetic opioid drugs (Comer & Cahill, 2019), particularly in North America where fentanyl is either taken intentionally or used to fortify heroin (Ciccarone, 2017; Kuczynska et al., 2018). The scale of this problem is alarming—in 2021, there were over 71,000 deaths in the United States related to synthetic opioids such as fentanyl, and this trend shows no sign of weakening (CDC & National Center for Health Statistics, 2022; Jannetto et al., 2019).

**FIGURE 1:**
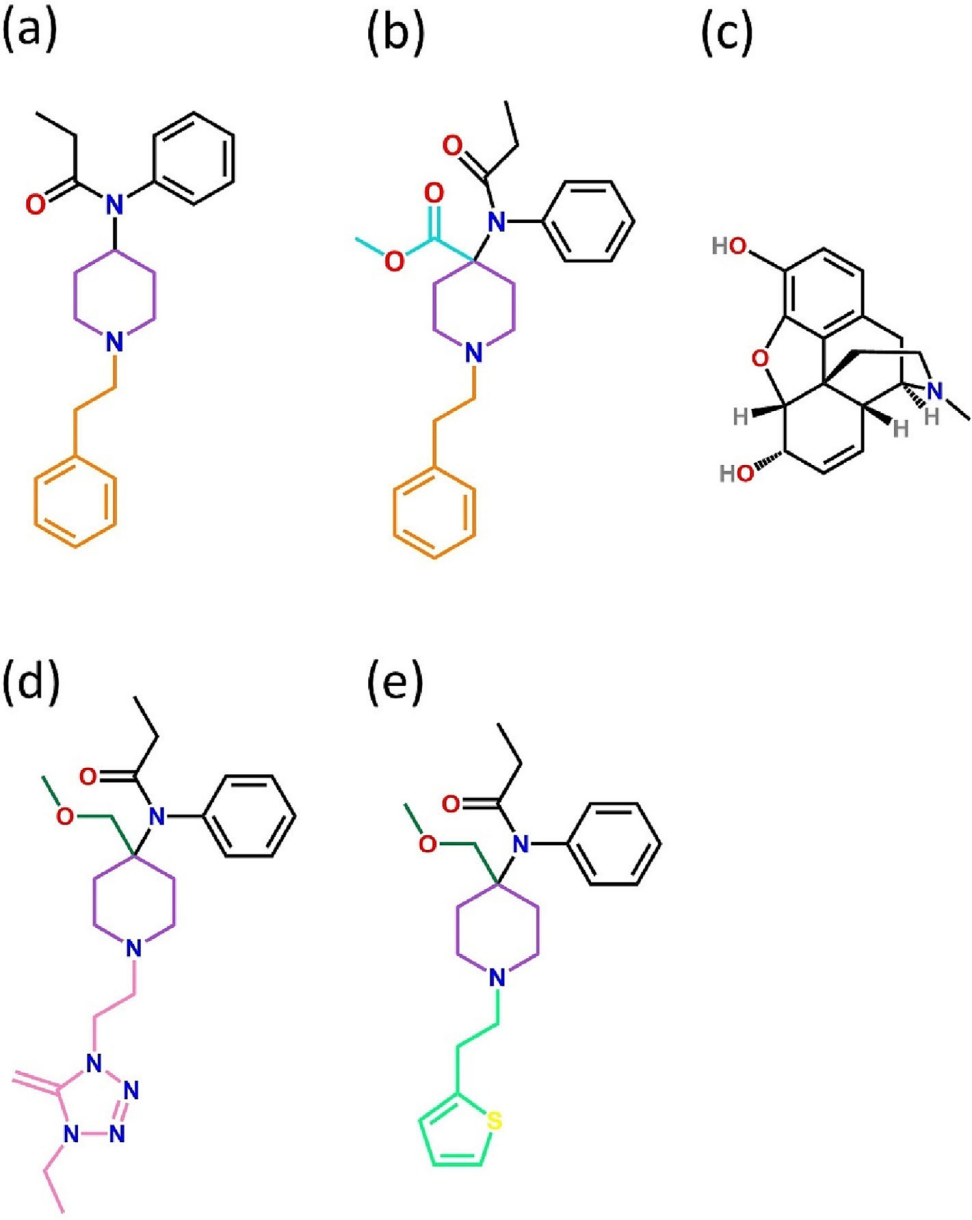
Molecular structures of four fentanyl ligands and morphine. Molecular structures of (a) fentanyl, (b) carfentanil, (c) morphine, (d) alfentanil and (e) sufentanil. Molecular groups making up the fentanyls are highlighted with different coloured lines; brown in (a) and (b), phenethyl group; purple in (a), (b), (d) and (e), piperidine group; black in (a), (b), (d) and (e), *N*‐phenyl‐propanamide; turquoise in (b), 4‐carbomethoxy; dark green in (d) and (e), methoxymethyl; pink in (d), *N*‐{1‐[2‐(4‐ethyl‐5‐oxo‐4,5‐dihydro‐1*H*‐1,2,3,4‐tetrazol‐1‐yl)ethyl]}; pale green in (e), *N*‐phenyl‐1‐[2‐(2‐thienyl)ethyl]. Nitrogen atoms are shown in blue, oxygen atoms are shown in red and sulphur atoms are shown in yellow.

Fentanyl and related drugs (known as “fentanyls”) differ structurally from classic morphinan opioids such as morphine (Figure 1c), with fentanyl consisting of a 4‐anilidopiperidine structure made up of an *N*‐phenyl‐propanamide group, a piperidine ring and an *N*‐phenethyl group (Figure 1a). Carfentanil has an additional carbomethoxy group on the fourth position of the fentanyl piperidine ring (Figure 1b). The fentanyl molecule is long and flexible and is more lipophilic in comparison to the more bulky and generally inflexible morphinan ligands (Figure 1c). Alfentanil and sufentanil both have an additional methoxymethyl group at the fourth position of the fentanyl piperidine ring; both ligands also have different groups in place of the phenethyl found in fentanyl (Figure 1d,e).

### 1.3 Aims

In this study, we explored the cell signalling profile of fentanyl and three related fentanyl‐like drugs (carfentanil, alfentanil and sufentanil) at the μ opioid receptor expressed in HEK293T cells, using BRET assays to assess both G protein activation and β‐arrestin 2 recruitment and trafficking assays to measure agonist‐induced cell surface loss of μ opioid receptors. In addition, electrophysiological recording was used to measure opioid‐evoked GIRK current and desensitisation at the endogenous receptor expressed in brain neurones. We used these assays to determine the relative potencies and efficacies of these ligands and also to determine whether any of these ligands might be biased relative to the prototypic μ opioid receptor agonist DAMGO 2([D‐Ala^2^, N‐MePhe^4^, Gly‐ol]‐enkephalin). Furthermore, we investigated the in silico interaction of fentanyl and carfentanil with the μ opioid receptor using molecular dynamics simulations. Our results indicated that of the fentanyls studied, carfentanil is an arrestin‐biased agonist at the μ opioid receptor and that carfentanil appears to interact with residues in the μ opioid receptor that are both common with, as well as distinct from, fentanyl.

## 2 METHODS

### 2.1 Drug compounds

For use in in vitro assays, drug stocks of DAMGO and fentanyl (Sigma‐Aldrich, Gillingham, Dorset, UK), morphine (MacFarlan Smith, Edinburgh, UK), alfentanil hydrochloride, sufentanil (Cayman Chemical, Ann Arbor, Michigan, USA) and β‐FNA (Tocris, Bristol, UK) were each dissolved in sterile de‐ionised water and stored at −20°C. Carfentanil (Cayman Chemical) was dissolved in a small amount of 100% DMSO before dilution in water. For cell signalling assays, drug stocks were further dissolved in phenol red‐free DMEM on the day of experiments. For electrophysiological experiments, all compounds or drugs were purchased from Sigma‐Aldrich except naloxone and Compound 101 (Hello Bio, Bristol, UK) and prazosin (Tocris). Drug stocks were prepared in de‐ionised water (or 100% DMSO for Compound 101) at 1000× the desired assay concentration and stored at −20°C. Drugs were diluted into the superfusing aCSF solution at 1:1000 ratio at the time of the experiment.

### 2.2 Cell culture

HEK293T cells were maintained at 37°C and 5% CO_2_ in 10 cm dishes, in Dulbecco's modified Eagle's medium (DMEM) supplemented with 10% fetal bovine serum and 2% penicillin/streptomycin. When 90% confluent, cells were transfected using lipofectamine 2000 in Opti‐MEM. For each dish of HEK293T cells, 5 ml of Opti‐MEM was added plus the additional 1 ml of DNA/lipofectamine mix. DNA constructs were added to dishes as follows: 3 μg Gαi1‐Renilla luciferase II, 3 μg GFP10‐Gγ2 and 3 μg HA‐rat μ opioid receptor for the G protein activation assay; 3 μg human μ opioid receptor‐Renilla luciferase II and 3 μg β‐arrestin 2‐GFP for the β‐arrestin 2 recruitment assay; 3 μg HA‐rat μ opioid receptor for the ELISA surface receptor loss (internalisation) assay. Cells were then incubated for a further 48 h before experimentation in either the BRET or ELISA assays.

### 2.3 Bioluminescence Resonance Energy Transfer assays

Concentration–response curves were generated using Bioluminescence Resonance Energy Transfer (BRET). On the day of the assay, transfected cells were washed and resuspended in phenol red‐free DMEM then aliquoted onto a 96‐well plate, 90 μl per well. BRET was carried out on a FLUOstar Omega plate reader (BMG LABTECH, Ortenberg, Germany), with measurements being made with 410 ± 80 nm and 515 ± 30 nm filters, the donor and acceptor wavelengths, respectively. All drug stocks were dissolved in water, coelenterazine 400a was dissolved in ethanol and further dilutions were achieved using phenol red‐free DMEM. Cells were incubated at 37°C with agonist drug for 2 min prior to the G protein dissociation measurement and 10 min prior to the measurement of β‐arrestin 2 recruitment; 5 μg·ml^−1^ coelenterazine 400a (VWR International, Lutterworth, UK) was then added and measurements taken at 5 s and 8 s post substrate injection. The ratio of the signal from the acceptor and donor was then calculated with the background signal subtracted. The assays were repeated to provide five independent experiments, with data being expressed either as % decrease in signal for the G protein dissociation assay or as raw data with basal subtracted for the β‐arrestin 2 recruitment assay.

Further BRET assays were carried out as described above, with an additional pre‐incubation of cells with the irreversible opioid receptor antagonist β‐funaltrexamine (β‐FNA). The β‐FNA was added at concentrations of either 0.1 μM or 0.01 μM for the G protein activation and β‐arrestin 2 recruitment assay, respectively, and incubated at 37°C for 1 h. Cells were then washed three times with PBS and plated for the BRET assay as above. Concentration–response data were analysed in Graphpad Prism 9.0, with curves fitted to a four parameter logistic model, apart from concentration–response data generated for cell surface receptor loss, which were fitted to a three parameter logistic model with the Hill slope constrained to 1, due to there being more variability in these data than in the other assays.

### 2.4 Enzyme linked immunosorbent assay

Drug‐induced cell surface loss of the μ opioid receptor was determined using an enzyme linked immunosorbent assay (ELISA) (Lowe et al., 2015). HEK293T cells were transfected as described in Section 2.2 with 3 μg of HA‐tagged μ opioid receptor or 3 μg pcDNA3.0 (empty vector) and incubated for 24 h, with cells then being plated onto poly‐l‐lysine coated 24‐well plates at 250,000 cells per well and incubated for a further 24 h. For experimentation, cells were serum starved for 15 min using Opti‐MEM, then agonist (morphine, DAMGO, fentanyl, carfentanil) or vehicle control was added and incubated for a further 1 h at 37°C. Cells were then fixed using 4% paraformaldehyde for 5 min before washing with TBS. Cells were then blocked for 45 min in 1% BSA, and following this, anti‐HA primary antibody (Biolegend, London, UK) was diluted 1:1000 and added for 1 h. Cells were then washed in TBS and blocked with 1% BSA for 15 min before addition of goat anti‐mouse IgG secondary antibody (Sigma‐Aldrich), diluted 1:1000, for 1 h. The resulting signal was detected using alkaline phosphatase incubation (Pierce PNPP substrate kit, Thermo Fisher, Swindon, UK), which bound to antibody‐tagged receptor; absorbance was read at 405 nm using a TECAN plate reader. Background absorbance was subtracted, and data are expressed as % cell surface loss of receptor.

### 2.5 Electrophysiological recordings

#### 2.5.1 Brain slice preparation

Male Wistar Rats (4 weeks old) (RRID:RGD_737929; originally purchased from Charles River then bred at the University of Bath for >10 years) were anaesthetized by intraperitoneal injection of 160 mg·kg^−1^ ketamine and 20 mg·kg^−1^ xylazine and then decapitated. The brains were rapidly removed and submerged in ice‐cold cutting solution composed of (in mM): 20 NaCl, 2.5 KCl, 1.6 NaH_2_PO_4_, 7 MgCl_2_, 85 sucrose, 25 d‐glucose, 60 NaHCO_3_ and 0.5 CaCl_2_ saturated with 95% O_2_/5% CO_2_. Horizontal brain slices (230 μm thick) containing the LC were prepared using a vibratome (DTK‐1000, DSK, Kyoto, Japan). Immediately after cutting, slices were incubated in warm (32°C) artificial cerebrospinal fluid (aCSF) containing (in mM): 125 NaCl, 2.5 KCl, 1.2 NaH_2_PO_4_, 1.2 MgCl_2_, 11.1 d‐glucose, 21.4 NaHCO_3_, 2.4 CaCl_2_ and 0.1 ascorbic acid, saturated with 95% O_2_/5% CO_2_ and were left to equilibrate for at least 1 h before recording. All animal care and experimental procedures were in accordance with the UK Animals (Scientific Procedures) Act 1986, the European Communities Council Directive (2010/63/EU) and the University of Bath ethical review document. Animal studies are reported in compliance with the ARRIVE guidelines (Percie du Sert et al., 2020) and with the recommendations made by the *British Journal of Pharmacology* (Lilley et al., 2020).

#### 2.5.2 Whole‐cell patch‐clamp electrophysiological recordings

Rat brain slices were transferred to a recording chamber and superfused with continuous flow (2.5 ml·min^−1^) of warm (32°C) aCSF. Whole‐cell recordings were made using recording electrodes (3–5 MΩ) containing an internal solution of (in mM): 115 potassium gluconate, 10 HEPES, 11 EGTA, 2 MgCl_2_, 10 NaCl, 2 MgATP and 0.25 Na_2_GTP, at pH 7.3 and with an osmolarity of 270 mOsm·L^−1^.

LC neurones were voltage‐clamped at −60 mV, with a correction made for a −12 mV junction potential. Activation of μ opioid receptor by opioids evoked GIRK currents, providing a real‐time measurement of μ opioid receptor activation which could be monitored continuously with the use of whole‐cell patch‐clamp recordings. All drugs were applied in the superfusing aCSF at known concentrations. In LC neurones, μ opioid receptors and α_2_‐adrenoceptors couple to the same population of GIRK channels (North & Williams, 1985). To control for cell‐to‐cell variability in GIRK current amplitude, currents were normalised to those evoked by 100 μM noradrenaline (NA), in the presence of 1 μM prazosin and 3 μM cocaine, in the same neurone (NA was applied to LC neurones both before and after the application of the opioid of interest, with prazosin and cocaine applied for 3 min prior to NA; opioid was applied after full reversal of the NA‐evoked current was observed, typically around 5 min after washout).

Concentration–response curves for DAMGO, morphine, fentanyl and carfentanil were generated in a cumulative manner. Each agonist concentration was superfused for at least 5 min prior to the addition of a higher concentration to the same cell. This was done to reduce the number of individual recordings, and accordingly the number of animals, required to complete this work. In the case of DAMGO and carfentanil, the highest studied concentrations of these agonists (10 μM and 300 nM, respectively) were examined separately due to desensitisation occurring at the preceding, lower concentrations in cumulative studies.

In our previous investigations of μ opioid receptor desensitisation in LC neurones, opioids were applied at receptor saturating concentrations (Groom et al., 2023; Rivero et al., 2012). Here, desensitisation of DAMGO, fentanyl and carfentanil‐evoked GIRK currents was assessed at approximately equi‐effective “just‐maximal” concentrations. This was due to safety concerns arising from the handling of large quantities of carfentanil. As such, agonist concentrations were standardised to approximately 30× the defined agonist EC_50_ in LC neurones (Table 1). Opioids were applied for at least 10 min, and the decline in the evoked GIRK current post‐peak response was tracked as a measure of receptor desensitisation. Naloxone (10 μM) was subsequently applied to antagonise fully opioid‐evoked currents and return responses to baseline. Where relevant, the GRK inhibitor Compound 101 (30 μM) was superfused for at least 20 min prior to, and during, application of opioid to LC slices (Groom et al., 2023; Lowe et al., 2015).

**TABLE 1:** Opioid agonist potency and maximum responses in different signalling assays.

(a) Potency				
	G‐protein activation log EC_50_ (M)	Opioid‐evoked GIRK current log EC_50_ (M)	β‐Arrestin 2 recruitment log EC_50_ (M)	Cell surface receptor loss log EC_50_ (M)
DAMGO	−6.48 ± 0.01	−7.29 ± 0.13	−5.70 ± 0.07	−6.14 ± 0.30
Morphine	−6.24 ± 0.14	−6.36 ± 0.03*	−5.96 ± 0.18	
Fentanyl	−6.79 ± 0.11	−7.42 ± 0.06	−6.41 ± 0.08	−7.61 ± 0.43*
Carfentanil	−8.11 ± 0.07*	−8.70 ± 0.11*	−8.50 ± 0.09*	−9.56 ± 0.18*
Alfentanil	−6.29 ± 0.04		−5.71 ± 0.07	
Sufentanil	−7.78 ± 0.17*		−7.49 ± 0.35	

(b) Maximum response				
	G‐protein activation (BRET signal % change from basal) E_max_	Opioid‐evoked GIRK current (I_m_ [% NA]) E_max_	β‐Arrestin 2 recruitment (raw BRET signal, basal subtracted) E_max_	Cell surface receptor loss (% change from vehicle) E_max_
DAMGO	14.2 ± 0.8	125 ± 2.4	1.1 ± 0.1	51.4 ± 8.4
Morphine	13.4 ± 0.5	85 ± 4.2*	0.4 ± 0.1*	
Fentanyl	14.7 ± 1.0	91 ± 5.8*	0.8 ± 0.1	44.3 ± 4.7
Carfentanil	16.6 ± 2.1	103 ± 9.4	1.3 ± 0.1	65.0 ± 9.6
Alfentanil	15.6 ± 1.4		1.1 ± 0.1	
Sufentanil	16.4 ± 0.7		1.0 ± 0.1	

*Note*: The table shows potency (Log EC_50_) and maximum responses (E_max_) values calculated for the concentration–response curves generated from the G protein activation assay, β‐arrestin 2 recruitment assay, brain slice GIRK current assay and the cell surface receptor loss assay. Log EC_50_ values and E_max_ values are given as mean ± SEM, n = 5. Log EC_50_ and E_max_ values were compared using a one‐way ANOVA with Dunnett's multiple comparison post‐test, all compared with DAMGO.

* Values were significantly different from the respective value for DAMGO (*P* < 0.05).

### 2.6 Bias calculations

Concentration–response curves generated in the G protein activation, β‐arrestin 2 recruitment, cell surface receptor loss and electrophysiological recording assays were fitted to the Black‐Leff operational model (Black & Leff, 1983) (Equation (1)) to generate Log(τ/K_A_) (Transduction Ratio) values (Kenakin et al., 2012; Kolb et al., 2022).

(1) $$E=\text{basal}+\frac{\left(E_{\max}-\text{basal}\right)\tau^{n}{\left[A\right]}^{n}}{\tau^{n}{\left[A\right]}^{n}+{\left(\left[A\right]+K_{A}\right)}^{n}}$$

where E is the response produced by the agonist, basal is the response without agonist, E_max_ is the maximum response from the tissue, τ is the operational efficacy (which is made up of multiple components within the system—receptor density, agonist intrinsic efficacy and the efficiency of receptor coupling to the effector), n represents the slope of the transducer function, [A] is the concentration of the agonist and K_A_ is the equilibrium dissociation constant. From the curve fitting, Log(τ/K_A_) values were generated for each drug in each assay; DAMGO was used as the reference agonist, and all drugs were first compared with DAMGO to give ΔLog(τ/K_A_) for a particular signalling pathway. The ΔLog(τ/K_A_) values were then compared for each drug between the signalling pathways to give ΔΔLog(τ/K_A_) values.

### 2.7 Calculating relative efficacy

For calculation of agonist efficacy using Furchgott's method, the approach used was that previously described (Dennis et al., 1992; Furchgott, 1966; Morey et al., 1998). Twenty parallel horizontal lines were drawn on the concentration–response curves for each agonist in the presence or absence of β‐FNA ([A′] and [A]) to give equieffective concentrations (i.e., points on the curves where the absolute response to the stimulus is the same), and log[agonist] (M) was recorded for both curves (see Figure S2). [A′] versus [A] was then plotted for each drug, for both G protein activation and β‐arrestin 2 recruitment (see Figure S3). These data were then fitted to Equation (2) (Dennis et al., 1992; Morey et al., 1998) using GraphPad prism 9.0.

(2) $$\left[A\right]=\frac{\left[A^{\prime }\right]q_{\text{functional}}K_{A}}{K_{A}+\left(1-q_{\text{functional}}\right)\left[A^{\prime }\right]}$$

In this equation, [A] and [A′] are the concentration of drug before and after treatment with β‐FNA, K_A_ is the equilibrium dissociation constant and q_functional_ is the fraction of receptors that are not inactivated by the presence of the β‐FNA. This gave values for K_A_ and q_functional_ for each drug, for each of the two assays. Using the K_A_ values, we then calculated occupancy for the agonist concentrations used in the concentration–response curves in the absence of β‐FNA, using Equation (3).

(3) $$p=\frac{\left[A\right]}{\left[A\right]+K_{A}}$$

Here, p is the fractional receptor occupancy at agonist concentration [A]. Finally, the % maximum response and % maximum occupancy versus agonist concentration were plotted on the same axes to allow the determination of the ratio K_A_/EC_50_ (see Figure 5). This also allowed the relationship between response and occupancy to be graphically represented (see Figure S4).

### 2.8 Molecular dynamics simulations

#### 2.8.1 Preparation of ligands

The 3D chemical structures of fentanyl and carfentanil were obtained from PubChem (pubchem.ncbi.nlm.nih.gov 2021). UCSF Chimera (Pettersen et al., 2004) was used to protonate the tertiary amine found in the structures. The ligand structures were parameterised using Antechamber and the general Amber force field (GAFF) (Wang et al., 2004). The ligands were solvated in a water box (TIP3P) (Jorgensen et al., 1983) with initial dimensions 45, 50 and 45 Å and with 0.15‐M NaCl. LEaP was used to generate Amber coordinate and topology files.

The system was minimised over 1000 steps with the ligand restrained then over 2500 steps without restraints. The system was then heated to 36.85°C (310^o^K) over 20 ps, then equilibrated under constant pressure for 100 ps. A 1μs trajectory for both fentanyl and carfentanil was generated, run under the ff14SB AMBER forcefield (Maier et al., 2015) and the general AMBER forcefield. The simulation had time steps of 2 fs with the trajectory being written every 100 ps. Temperature was controlled using the Langevin thermostat and pressure was controlled using the anisotropic Berendsen barostat. The 1 μs trajectory of each ligand was analysed using CPPTRAJ (Roe & Cheatham, 2013) to strip the water and ions and give 10,000 conformations of each ligand.

#### 2.8.2 Preparation of the receptor structure

The X‐ray crystal structure of the inactive μ opioid receptor bound to the antagonist β‐FNA was originally from the protein databank (PDB: 4DKL) (Manglik et al., 2012), a modified version of this receptor was used with the T4 lysozyme fusion protein removed and the third intracellular loop rebuilt using a homologous loop (Sutcliffe et al., 2017).

The X‐ray crystal structure of the active μ opioid receptor bound to agonist BU72 was also obtained from the protein databank (PDB: 5C1M) (Huang et al., 2015). The ions and lipids not part of the ligand or protein were removed from the structure, and bound nanobody Nb39 was also removed. The structure of helix 8 (H8) was rebuilt by overlaying with the H8 of the inactive‐state receptor structure; N‐terminal residues were also removed from the structure down to MET65^1.29^ so that the active‐state structure matched the inactive‐state.

#### 2.8.3 Docking into the μ‐opioid receptor using the Bristol University Docking Engine

Fentanyl and carfentanil were each docked into the μ opioid receptor structures using the Bristol University Docking Engine (BUDE) (McIntosh‐Smith et al., 2015). Each of the 10,000 poses generated from the water box simulations were docked independently. The docking was confined to a box of 15 × 15 × 15 Å centred over the orthosteric binding pocket of each of the μ opioid receptor structures. BUDE searched this specified space for the lowest energy pose for each conformation, and the space was explored using x, y, z movements and full rotation of the ligand, using BUDE's genetic algorithm. This gave 10,000 distinct docked conformations of fentanyl and carfentanil, which were ordered by energy. The 50 lowest energy poses for each ligand in each of the two μ opioid receptor structures were assessed in Chimera, determining the proximity to the conserved residue Aspartate 147 (Li et al., 1999; Surratt et al., 1994) (ASP147^3.32^; superscript numbers follow the Ballesteros–Weinstein numbering system for GPCR residues; Ballesteros & Weinstein, 1995) as well as any distinct clusters of poses.

#### 2.8.4 Simulation of the ligand–receptor complex

Ligand–receptor complexes were generated for two distinct poses per ligand (fentanyl and carfentanil), in each of the inactive and active state crystal structures of the μ opioid receptor. The ligand–receptor complexes for each pose were inserted into a bilayer containing POPC:POPE:cholesterol at a ratio of 2:2:1 using CHARMM‐GUI (Jo et al., 2008). This was then placed in a simulation box containing TIP3P water and 0.15‐M NaCl with initial dimensions of 90 × 110 × 90 Å. LEaP, an Amber utility, was used to generate coordinate and topology files for each ligand–receptor complex. For each system, the workflow is as follows: The system was first minimised over 10,000 steps, then heated in two steps, from 0 K to 100 K over 2500 steps, and then from 100 K to 310 K over 50,000 steps. The system's periodic boundary condition dimensions were then equilibrated in 10 steps each lasting 500 ps, the steps are consecutive and each is restarted from the point the previous step finished. Initial 125‐ns trajectories for each complex were generated using the ff14SB (Maier et al., 2015) and lipid14 (Dickson et al., 2014) AMBER force fields. The simulations had time steps of 2 fs with the trajectories being written every 100 ps. Temperature for the simulations was controlled using the Langevin thermostat, and pressure was controlled using the anisotropic Berendsen barostat. To generate 1‐μs datasets for each ligand, in both receptor structures, seven evenly spaced timepoints were used along the initial 125‐ns trajectory, and the trajectory frame at each of these timepoints was then used to start another 125‐ns trajectory, with new initial velocities; this was combined to give 8× 125‐ns simulations. This was carried out for two poses for both fentanyl and carfentanil in both the inactive and active state μ opioid receptor.

#### 2.8.5 Analysis of trajectories and generation of simulation images

Molecular graphics were generated using UCSF Chimera (Pettersen et al., 2004). All analysis was performed using CPPTRAJ (Roe & Cheatham, 2013), and plots were made in Microsoft Excel.

### 2.9 Data analysis and statistics

The data and statistical analysis comply with the recommendations of the *British Journal of Pharmacology* on experimental design and analysis in pharmacology (Curtis et al., 2022). The data analysis including statistical testing was carried out in Graphpad Prism 9.0. Studies were designed to generate groups of equal size, with drug additions randomised as much as possible; however, blinded analysis was not undertaken although data were processed in spreadsheets. All BRET and ELISA values are expressed as mean ± S.E.M., where n = 5 independent experiments; the BRET and ELISA experiments were run in duplicate and triplicate (technical replicates) respectively and averaged to form an individual repeat (n). Statistical analysis was undertaken only for studies where each group size was n = 5. For all assays, data points were excluded where their value was >3× S.D. different from the mean of the other values. For the BRET and ELISA assays, Log EC_50_ and E_max_ values ± S.E.M. were obtained by fitting curves from each experiment in GraphPad Prism 9.0 individually and then combining to give mean values with calculated ±S.E.M. Note that it was not possible to fit the data from each individual morphine β‐arrestin 2 BRET experiment to a four‐parameter model as this provided ambiguous results in GraphPad Prism; therefore, the morphine β‐arrestin 2 values in Table 1 were obtained by fitting each individual experiment to a three‐parameter curve, which produced unambiguous values of Log EC_50_ in each case. One‐way ANOVA with Dunnett's multiple comparisons post‐hoc test was used to test for statistical significance between Log EC_50_ and E_max_ values for agonists calculated from concentration–response curves, extent of desensitisation of opioid‐activated GIRK currents and also for differences in ΔΔLog(τ/K_A_) values, using the values obtained for DAMGO as a reference to compare to. Statistical significance was defined as *P* < 0.05, whilst an *F* value of 4 was used for the ANOVA to indicate the required statistical significance. β‐Arrestin recruitment curves are given as raw data with the basal removed, G protein activation curves are given as % change from basal and cell surface receptor loss is given as % change from vehicle; none of these curves were normalised to a drug standard, only to their own basals. Addition of cells to the 96‐well or 24‐well plates was performed randomly pre‐experiment, and for both the BRET and ELISA assays, the overall order in which agonists were added to the plates as well as the order of the concentrations of each drug added across the plate were randomised for each experiment. Accordingly, the location of the basal or vehicle controls was different for each experiment. Blinding was not undertaken in these experiments firstly due to the complexity of the plate assays with multiple agonists and concentrations and secondly because of safety concerns with the use of concentrated solutions of highly potent and potentially harmful opioid drugs. For calculation of relative efficacy, 20 approximately equally spaced horizontal lines were drawn between ascending portions of the log agonist‐concentration response curves in the absence and presence of β‐FNA. For LC electrophysiology experiments, each separate repeat of an experimental condition (n) represents experiments in brain slices derived from separate animals. If any data points from these experiments were obtained with n numbers less than 5, then this is clearly stated in the figure legend. Both data collection and analysis were conducted unblinded for practical reasons, as the experimenter had to apply the pharmacological agents themselves, apart from safety concerns with the handling of fentanyls. The only normalisation of data was undertaken with the electrophysiological data, to control for cell‐to‐cell variability, where currents were normalised to those evoked by 100‐μM noradrenaline in the same neurone. Statistical significance of differences in EC_50_, E_max_ and differences in ΔΔLog (τ/K_A_) values was determined by one‐way ANOVA with Dunnett's multiple comparisons post hoc test to determine differences from DAMGO, as described above for the cell signalling assays. Post‐hoc tests were run only if F achieved P<0.05 and there was no significant variance inhomogeneity.

### 2.10 Nomenclature of targets and ligands

Key protein targets and ligands in this article are hyperlinked to corresponding entries in http://www.guidetopharmacology.org, and are permanently archived in the Concise Guide to PHARMACOLOGY 2021/22 (Alexander et al., 2021).

## 3 RESULTS

### 3.1 Signalling of fentanyls through the G protein and β‐arrestin pathways

**FIGURE 2:**
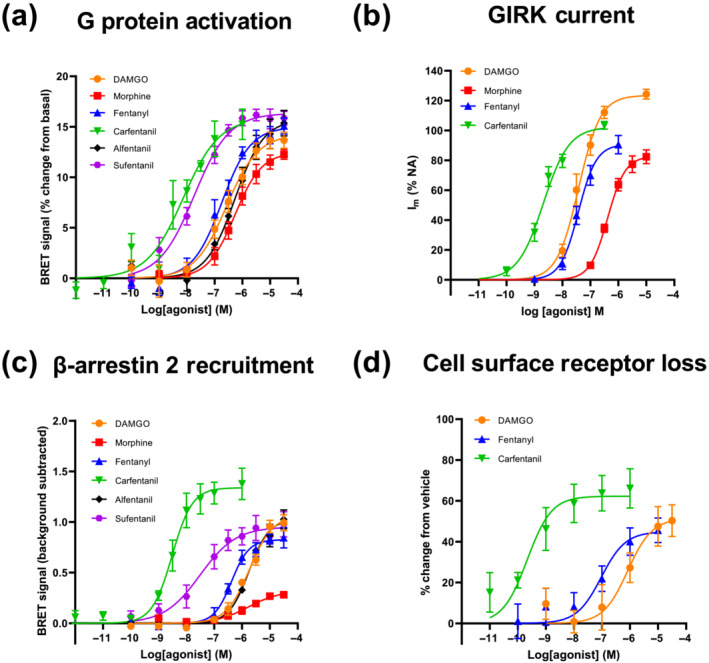
Log concentration–response data for agonists activating the μ opioid receptor. Log concentration–response curves for (a) opioid‐induced G protein activation, measured by BRET; (b) opioid‐evoked GIRK currents, measured by whole cell patch clamp electrophysiological recording; (c) opioid‐induced β‐arrestin 2 recruitment, measured by BRET; (d) opioid‐induced cell surface μ opioid receptor loss, measured by ELISA assay. BRET data in (a) and (c) are expressed as the mean of duplicate readings, n = 5; G protein activation is expressed as a % change from basal, β‐arrestin recruitment is expressed as raw BRET ratio with the background subtracted. Measures of GIRK current (I_m_) in (b) are normalised to the maximal α_2_‐adrenoceptor‐mediated current evoked by noradrenaline (NA; 100 μM) in the same cell, n = 5 for data points apart from the −5 and −5.5 log concentrations for morphine where n = 4, and for the −8.5 and −6.5 log concentrations for carfentanil where n = 3. Cell surface receptor loss is expressed as the mean of triplicate readings, n = 5, with data expressed as % change from vehicle control. Error bars are ±SEM. In all cases, the bottoms of the concentration–response curves were constrained to a response level of zero. Data were fitted to either a three‐parameter (a, b, c) or four‐parameter (d) logistic model using Graphpad Prism 9.0.

The ability of four fentanyls (fentanyl, carfentanil, alfentanil and sufentanil) to activate the μ opioid receptor and signal through G proteins was assessed using Gi protein activation BRET (Figure 2a). Here, an increase in BRET signal, expressed as the percentage change from basal, indicates dissociation of G protein subunits (Giα‐RLucII and Gγ‐GFP10) and thus increased G protein activation (Denis et al., 2012). The prototypical μ opioid receptor peptide agonist DAMGO and the morphinan agonist morphine were assessed alongside the fentanyls. Morphine, fentanyl and alfentanil activated Gi protein with similar potency to DAMGO whilst sufentanil and carfentanil displayed 31‐fold and 42‐fold higher potency, respectively, compared with DAMGO (Figure 2a and Table 1). All four fentanyls and DAMGO signalled with a similar E_max_ for G protein activation (Figure 2a and Table 1).

The potency and efficacy of fentanyl, carfentanil, DAMGO and morphine at the μ opioid receptor was further assessed in a setting of physiological μ opioid receptor expression: rat LC neurons. In these neurons, μ opioid receptors couple via G proteins to GIRK channels, meaning that opioid‐induced GIRK currents recorded through whole‐cell voltage‐clamped electrophysiological recordings could be used to monitor μ opioid receptor activity in real‐time (Figure 2b). Whilst fentanyl and DAMGO displayed equivalent potency in LC neurones, carfentanil exhibited a 25‐fold higher potency, mirroring our findings from G protein activation assays in a recombinant expression system (Figure 2a). In rat LC neurones, the fitted E_max_ values for fentanyl and morphine were significantly lower than that of DAMGO (Table 1), suggesting a difference in intrinsic efficacy not observed in the G protein activation BRET assay (Figure 2a); this is explored further in Section 3.3. The peak GIRK current evoked by carfentanil was not significantly altered following inhibition of GRK by Compound 101 (30 μM) (see Figure S5), suggesting that we can discount the possibility of the peak response being underestimated by rapid receptor desensitisation occurring before the plateau of the peak carfentanil‐evoked current is reached.

The ability of the fentanyls to recruit β‐arrestin 2 to the μ opioid receptor was measured using a β‐arrestin 2 recruitment BRET assay (Figure 2c). Here an increase in the BRET signal indicates increased β‐arrestin 2 recruitment to the μ opioid receptor (β‐arrestin 2‐GFP10 and μ opioid receptor‐RlucII) (Molinari et al., 2008). In this assay, and of particular interest, carfentanil displayed very high potency, 625‐fold higher than DAMGO (Figure 2c and Table 1). On the other hand morphine displayed a significantly lower E_max_ for β‐arrestin 2 recruitment compared with the other drugs in the assay, indicating lower efficacy for this response (Figure 2c and Table 1).

DAMGO‐, fentanyl‐ and carfentanil‐induced cell surface loss of μ opioid receptor was assessed using an ELISA assay with HA‐tagged receptor following 1 h exposure to the opioid agonists (Lowe et al., 2015) (Figure 2d). Agonist‐induced cell surface loss of receptor is a β‐arrestin‐mediated process considered to largely represent receptor internalisation (Lowe et al., 2015). Similar to the β‐arrestin 2 recruitment results in the BRET assay, carfentanil induced very potent cell surface loss of the μ opioid receptor, being approximately 2600‐fold more potent than DAMGO (Figure 2d and Table 1). DAMGO‐induced cell surface loss of μ opioid receptor was comparable to previously published data (Johnson et al., 2006; Lowe et al., 2015). Morphine was not studied here as it was previously shown that morphine‐induced cell surface receptor loss is negligible compared with DAMGO (Bailey et al., 2003; Johnson et al., 2006; Keith et al., 1996). It should also be noted that whereas the β‐arrestin 2 recruitment assays were carried out using the human μ opioid receptor, the other assays including cell surface receptor loss were undertaken with the rat receptor. Nevertheless, the relative potencies of the agonists in the β‐arrestin 2 and cell surface receptor loss assays were similar, suggesting the rat and human receptors respond to agonist in the same manner (Figure 2c,d).

Carfentanil's high potency for both β‐arrestin 2 recruitment and cell surface loss of receptor support the idea that carfentanil displays β‐arrestin bias.

### 3.2 Assessing the signalling bias of fentanyl ligands

**FIGURE 3:**
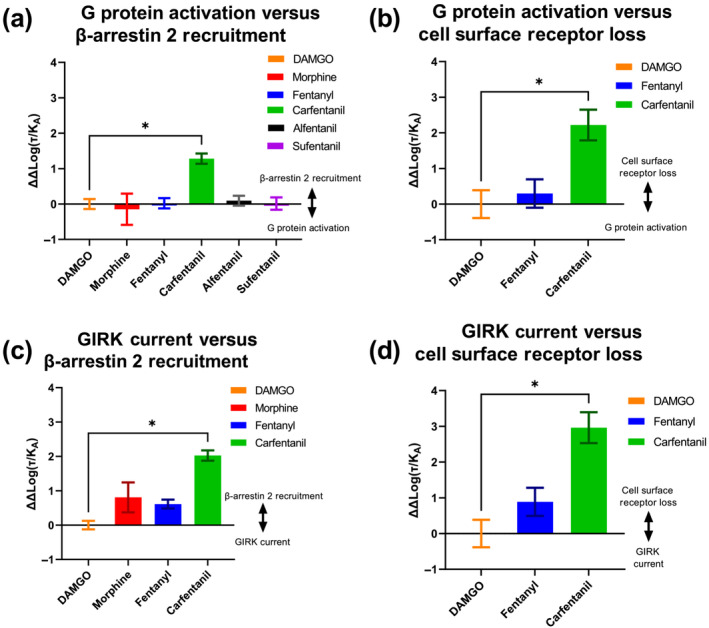
Ligand bias calculations based on the experimental data in Figure 2. (a) Graph of ΔΔLog (τ/K_A_) values generated by comparing data from the G protein activation BRET assay and the β‐arrestin 2 recruitment BRET assay. DAMGO was used as the reference agonist. Carfentanil was significantly β‐arrestin 2 biased compared with DAMGO; none of the other agonists tested were biased compared with DAMGO. (b) Graph of ΔΔLog (τ/K_A_) values generated by comparing data from the G protein activation BRET assay and the cell surface loss of the μ opioid receptor assay. DAMGO was used as the reference agonist and carfentanil was significantly biased towards cell surface receptor loss. (c) Graph of ΔΔLog (τ/K_A_) values generated by comparing data from the GIRK current assay and the β‐arrestin 2 recruitment BRET assay. (d) Graph of ΔΔLog (τ/K_A_) values generated by comparing data from the GIRK current assay and the cell surface loss of μ opioid receptor assay. Statistical significance was determined by one‐way ANOVA with Dunnett's multiple comparison post hoc test with DAMGO as the reference agonist (*P* < 0.05, *). In none of the four comparisons in (a)–(d) was fentanyl significantly different from the reference agonist DAMGO.

Bias was calculated by fitting the concentration–response curves from all four in vitro assays to the operational model of agonist action (Black & Leff, 1983). From this, Log(τ/K_A_) (Transduction Ratio) values were obtained, followed by ΔLog(τ/K_A_) values compared with DAMGO as the reference agonist, and then ΔΔLog(τ/K_A_) values to compare signalling through the different pathways for each agonist (Kenakin et al., 2012; see Table S1). When comparing the G protein BRET and β‐arrestin 2 BRET assays, carfentanil displayed strong β‐arrestin 2 bias relative to DAMGO (Figure 3a), whereas the other four agonists did not display any bias. Further bias calculations were undertaken by comparing agonist‐induced G protein BRET responses to cell surface loss of μ opioid receptor (Figure 3b), GIRK current from brain slices to β‐arrestin 2 BRET recruitment (Figure 3c), and GIRK current from brain slices to cell surface loss of μ opioid receptor (Figure 3d). In each case, relative to DAMGO, carfentanil displayed β‐arrestin bias when comparing responses mediated by β‐arrestin 2 (β‐arrestin 2 recruitment by BRET and cell surface loss of μ opioid receptor) with G protein‐mediated responses (G protein activation by BRET and GIRK current activation). Therefore, relative to the reference agonist DAMGO, carfentanil was the only one of the five agonists studied that displayed statistically significant β‐arrestin 2 bias.

Bias was also calculated between cell surface receptor loss and β‐arrestin 2 recruitment, as well as between G protein activation and GIRK current. The calculated ΔΔLog(τ/K_A_) values for the comparison of cell surface receptor loss and β‐arrestin 2 recruitment revealed no significant difference between the two pathways, relative to DAMGO (Figure S1a). However, the calculated ΔΔLog(τ/K_A_) for the comparison of G protein activation and GIRK current revealed that morphine, fentanyl and carfentanil are all significantly different from DAMGO in the direction of G protein activation (Figure S1b). This is probably due to DAMGO having a higher E_max_ for GIRK current relative to the other agonists, which is not observed in the G protein activation BRET assay (Figure 2a,b).

### 3.3 Determining the efficacy of fentanyl and other ligands by decreasing receptor reserve

**FIGURE 4:**
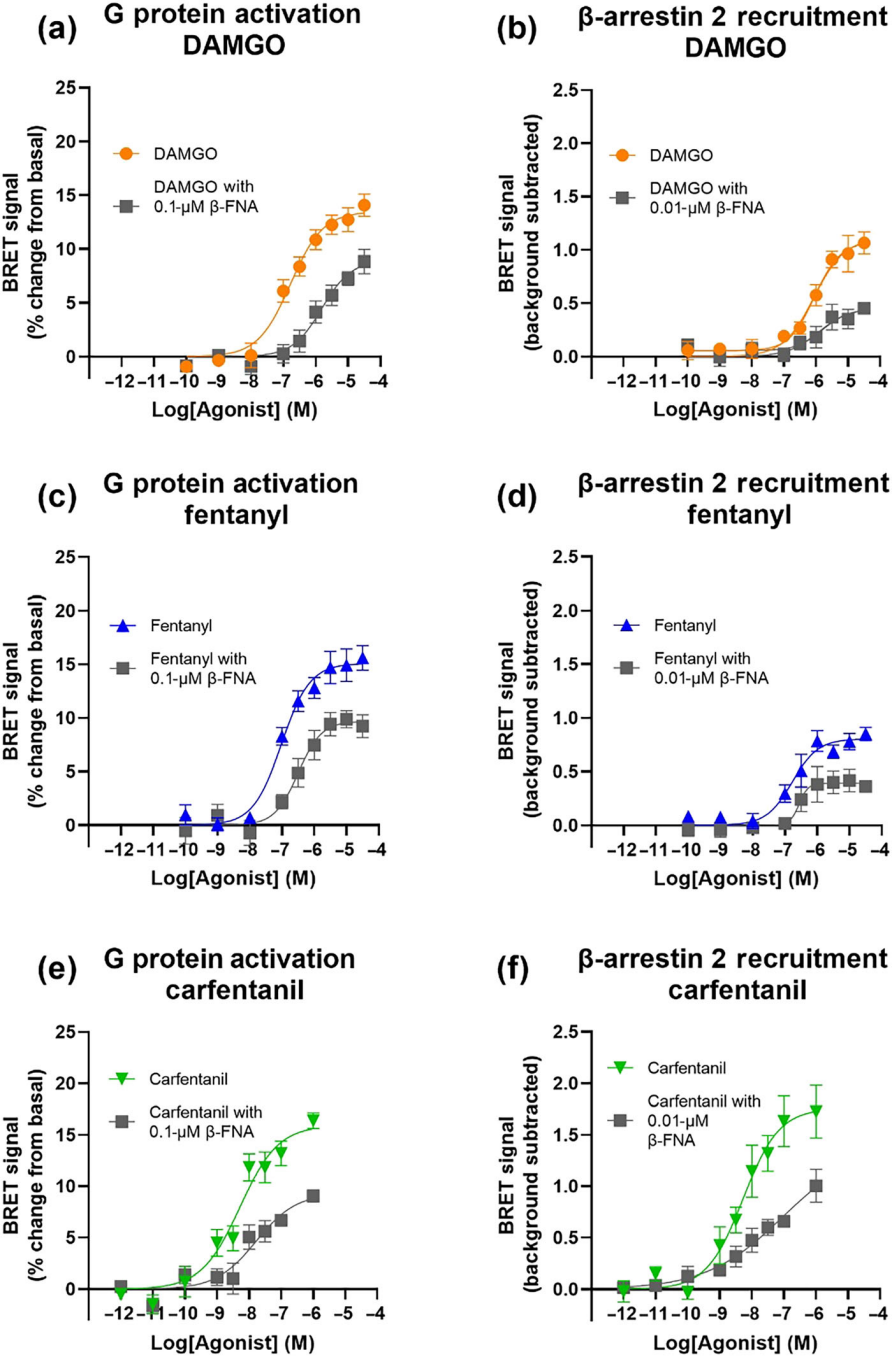
Log concentration–response curves for G protein activation or β‐arrestin 2 recruitment in the presence or absence of receptor inactivation. Shown are data from BRET assays for DAMGO (a, b), fentanyl (c, d) and carfentanil (e, f), in the absence (colour) or presence (dark grey) of pretreatment with the irreversible antagonist β‐FNA (0.1 μM for G protein activation and 0.01 μM for β‐arrestin 2 recruitment). BRET assays used are G protein activation (a, c, e) and β‐arrestin 2 recruitment (b, d, f). Data are expressed as mean of duplicate readings, n = 5. Error bars are ±SEM.

The relative efficacies of DAMGO, fentanyl and carfentanil were determined for G protein activation and β‐arrestin 2 recruitment by generating concentration–response curves in the presence or absence of the irreversible μ opioid receptor antagonist β‐FNA (Figure 4a–f), in order to decrease receptor reserve. For all three drugs, decreasing the receptor reserve caused a decrease in the Emax and varying degrees of rightward shift of the concentration–response curve. In order to quantify the efficacy of each drug for the two pathways we used a previously employed version of the Furchgott method (Dennis et al., 1992; Furchgott, 1966; Morey et al., 1998).

**FIGURE 5:**
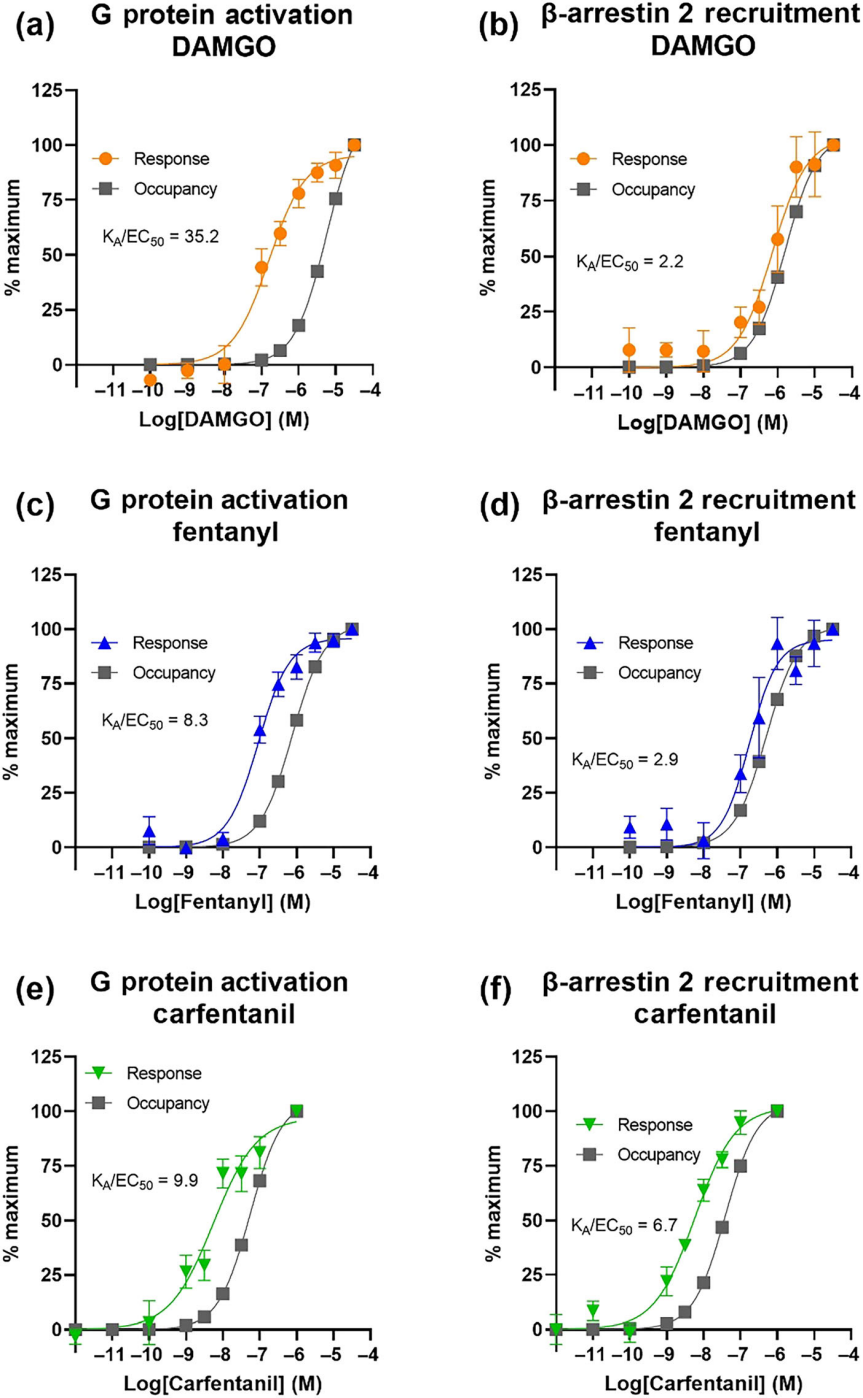
Log concentration–response and log concentration–occupancy curves. Graphs show log concentration–response curves (colour) and log concentration–occupancy curves (dark grey) for DAMGO (a, b), fentanyl (c, d) and carfentanil (e, f), for G protein activation (a, c, e) and β‐arrestin 2 recruitment (b, d, f) BRET assays. The log concentration–response curves are the same data as in Figure 4, normalised to the % maximum response for each agonist in each assay, data are mean ± SEM. Log concentration–occupancy curves were generated from Equation (3) and expressed as % maximum for each agonist. EC_50_ and K_A_ values were read off from each of the response and occupancy curves, respectively, and the ratio of the two is given on each graph.

Using the concentration–response curves for the three drugs in the presence or absence of β‐FNA we were able to calculate agonist affinity as described in the Methods (see also Figures S2 and S3) and hence construct agonist log concentration–response and log concentration‐occupancy plots (Kelly, 2013) (Figure 5). From this we were able to calculate K_A_/EC_50_ for the three agonists, with a greater ratio of K_A_/EC_50_ being indicative of greater receptor reserve and hence efficacy of that agonist (Table 2). It should be noted that the K_A_ values here have been generated from whole cell experiments with ~120‐mM extracellular sodium, which is known to affect the affinity of high efficacy agonists (Kelly, Sutcliffe, et al., 2023); these K_A_ values can therefore not be compared directly to those obtained from radioligand binding assays carried out in the absence of sodium.

**TABLE 2:** Summary table of values calculated from data in Figures 4 and 5.

		K_A_ (nM)	K_A_/EC_50_	Occupancy required for 50% of response	q_functional_
G protein activation	DAMGO	5596 (95% CI [4564, 6860])	35.2	3.4	0.06 (95% CI [0.05, 0.07])
	Fentanyl	753 (95% CI [531, 1055])	8.3	11.5	0.21 (95% CI [0.18, 0.25])
	Carfentanil	54 (95% CI [41, 71])	9.9	9.4	0.11 (95% CI [0.09, 0.13])
β‐arrestin 2 recruitment	DAMGO	1585 (95% CI [1077, 2245])	2.2	30.6	0.26 (95% CI [0.21, 0.32])
	Fentanyl	499 (95% CI [215, 1039])	2.9	27.8	0.31 (95% CI [0.26, 0.46])
	Carfentanil	39 (95% CI [23, 61])	6.7	12.4	0.13 (95% CI [0.09, 0.18])

*Note*: K_A_ is the equilibrium dissociation constant, calculated by plotting A′ versus A for each drug and fitting to Equation (2). K_A_/EC_50_ is the ratio of the K_A_ calculated from the receptor occupancy curve, and the EC_50_ calculated from the response curve for each agonist (see Figure 2a,c; see Table 1). The occupancy required for 50% of the maximum response was calculated by plotting occupancy against response, see Figure S4. The parameter q_functional_ refers to the fraction of receptors that are not inactivated by the presence of the β‐FNA. Confidence intervals were generated for the K_A_ and q_functional_ values and are represented as (95% CI [lower limit, upper limit]).

In the G protein activation assay, DAMGO displayed the largest K_A_ (5596 nM) indicating relatively low affinity for the receptor and a K_A_/EC_50_ ratio of 35.2. Fentanyl and carfentanil displayed similar K_A_/EC_50_ ratios (8.3 and 9.9, respectively) indicating similar efficacy to each other but lower than that of DAMGO (Figure 5a,c,e). Carfentanil displayed the lowest K_A_ (54.4 nM) suggesting high affinity for the receptor. Thus, DAMGO had the highest efficacy of the three drugs for G protein activation, which is in agreement with the higher E_max_ for DAMGO displayed in the endogenous system (Figure 2b).

In the β‐arrestin 2 recruitment assay, of the three agonists, carfentanil again had the lowest K_A_ for the μ opioid receptor (39 nM). DAMGO and fentanyl displayed similar K_A_/EC_50_ ratios for β‐arrestin 2 recruitment (2.2 and 2.9, respectively), indicating similar efficacy (Figure 5b,d,f).

Carfentanil displayed a K_A_/EC_50_ ratio approximately three times higher than DAMGO and fentanyl (6.7), indicating that carfentanil has the highest efficacy of the three drugs for β‐arrestin 2 recruitment. This is in line with carfentanil's β‐arrestin bias reported above.

### 3.4 Measuring desensitisation of opioid‐evoked GIRK currents

**FIGURE 6:**
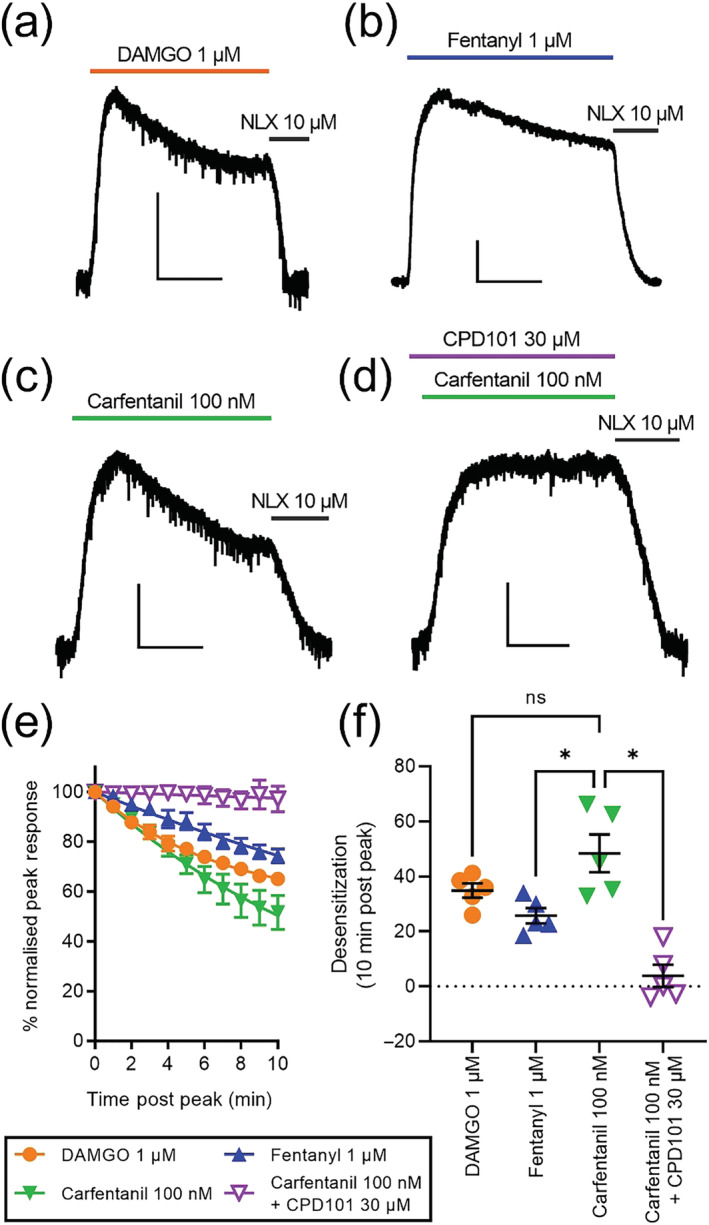
Electrophysiological recordings of opioid‐activated GIRK currents from rat locus coeruleus neurones. Traces in (a), (b) and (c) show individual recordings from different neurones of opioid‐evoked GIRK currents from 1 μM DAMGO, 1 μM fentanyl and 100 nM carfentanil. Agonist drug addition was followed by 10 μM of the μ opioid receptor antagonist naloxone (NLX). (d) Trace shows 100‐nM carfentanil response in the presence of 30 μM of the GRK inhibitor Compound 101 (CPD101), followed by 10 μM naloxone. Scale bars in (a)–(d) represent 50 pA and 300 s. (e) Graph of decay of opioid‐evoked GIRK current recorded for 10 min in the presence of drug; 1 μM fentanyl, 1 μM DAMGO, 100 nM carfentanil and 100 nM carfentanil in the presence of 30 μM Compound 101. Data points were fitted to a one‐phase decay model; error bars show ±SEM, n = 5. (f) Graph showing desensitisation after 10 min post peak for DAMGO, fentanyl, carfentanil and carfentanil in the presence of Compound 101, datapoints from five individual experiments are shown, mean ± SEM is given by black bars. Carfentanil‐induced desensitisation is significantly different from fentanyl‐induced desensitisation and carfentanil in the presence of Compound 101 (*P* < 0.05, *; one‐way ANOVA with Dunnett's multiple comparisons post hoc test).

The potential functional consequence of carfentanil's β‐arrestin 2 bias and high efficacy for β‐arrestin 2 on its ability to induce receptor desensitisation was investigated using endogenous μ opioid receptors in rat LC neurones. Application of a just maximal concentration of DAMGO (1 μM, Figure 6a), fentanyl (1 μM, Figure 6b) or carfentanil (100 nM, Figure 6c) evoked GIRK currents that exhibited different degrees of desensitisation over the 10 min drug application period (Figure 6e). At 10 min post response peak, the extent of μ opioid receptor desensitisation induced by carfentanil was significantly greater than that by fentanyl, but there was no significant difference between the desensitisation induced by DAMGO and carfentanil (Figure 6f).

In order to investigate the mechanism of carfentanil‐induced μ opioid receptor desensitisation in LC neurones, slices were preincubated with the GRK inhibitor Compound 101 (30 μM) for 20 min prior to and then during carfentanil application (Figure 6d,f). Carfentanil‐induced μ opioid receptor desensitisation was abolished in the presence of Compound 101, suggesting that the GRK/β‐arrestin 2 mechanism mediates carfentanil‐induced desensitisation in this system (Figure 6e,f; see also Figure S5).

### 3.5 Docking and molecular dynamics of fentanyl and carfentanil at the μ opioid receptor

**FIGURE 7:**
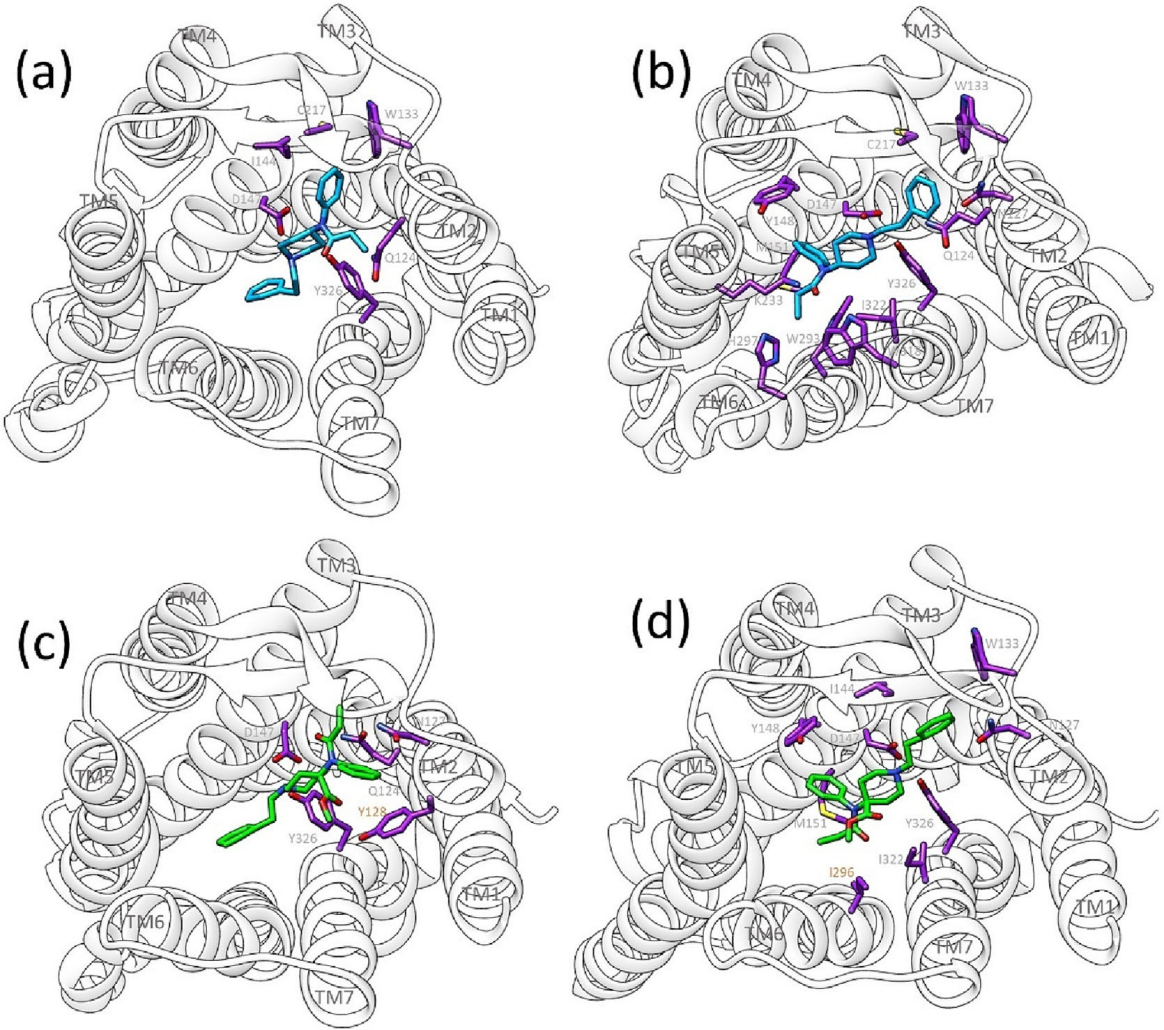
Images showing the final frame of 1μs molecular dynamics simulations of fentanyl and carfentanil in differing orientations/poses. The images are taken from above the receptor, looking down into the membrane. (a) Fentanyl pose1, phenethyl towards intracellular side of receptor; (b) fentanyl pose 2, phenethyl towards extracellular side of receptor; (c) carfentanil pose 1, phenethyl towards intracellular side of receptor; (d) carfentanil pose 2, phenethyl towards extracellular side of receptor. Fentanyl is shown in blue, and carfentanil is shown in green. Receptor residues shown are those that lie within 4 Å of the ligand for >50% of the simulation, these are shown in purple and have been labelled for each image. In (c) and (d), the two residues (Y128 and I296 respectively) that are within 4 Å of carfentanil's 4‐carbomethoxy group are indicated by orange lettering/numbering. Oxygen atoms are shown in red, nitrogen atoms are shown in blue and sulphur atoms are shown in yellow. Transmembrane domains have been labelled.

To further explore the interaction of fentanyl and carfentanil with the μ opioid receptor at the molecular level, each ligand was docked into the inactive‐state crystal structure of the μ opioid receptor to determine energetically favourable binding poses. Initial 125‐ns MDs were generated of potentially stable poses, and these simulations were analysed for ligand stability and ligand proximity to ASP147^3.32^. Following this, 1 μs of all atom MD simulation was generated for stable ligand binding poses of fentanyl and carfentanil in the μ opioid receptor, which was embedded in a phospholipid bilayer. For both fentanyl and carfentanil, two potential pose orientations were determined: with the phenethyl moiety positioned either towards the intracellular (pose1; Figure 7a,c; also Figure S7a,c) or extracellular (pose2; Figure 7b,d; also Figure S7b,d) side of the receptor. In both poses, fentanyl and carfentanil displayed stable binding in MD simulations, as demonstrated by stable RMSD (Figure S6) and a <4 Å distance from the protonated amine of the ligand to the conserved ASP147^3.32^ residue of the receptor (superscript numbers follow the Ballesteros–Weinstein numbering system for GPCR residues) (Ballesteros & Weinstein, 1995) (Figure S6).

Each ligand, in its two potential binding orientations, formed both common and unique interactions with the residues in the orthosteric binding site of the μ opioid receptor (Table S2). Potential residue contacts were calculated from the 1‐μs MD simulation and residues that were within a 4 Å distance of the ligand along with the % of the simulation time that they were within that 4 Å distance were highlighted (Table S2).

In the pose1 orientation, fentanyl and carfentanil made contacts with residues primarily in TM2 and TM3 with an additional conserved interaction with TYR326^7.43^ in TM7. In pose1, fentanyl formed interactions in ECL1 and ECL2 not observed for carfentanil in pose1.

More residue contacts were formed by fentanyl and carfentanil in the pose2 orientation compared with the pose1 orientation. In the pose2 orientation, both fentanyl and carfentanil interacted with residues in TM2, ECL1, TM3 and TM7. In this orientation, fentanyl made further contacts with TM5 and TM6, which were not observed for carfentanil.

Structurally, carfentanil differs from fentanyl only in its 4‐carbomethoxy moiety (Figure 1), and from our MD simulations, we determined that this moiety interacts with tyrosine128^2.64^ (TYR128^2.64^; 62% of the MD simulation) or isoleucine296^6.51^ (ILE296^6.51^; 32% of the MD simulation) in either the pose1 or pose2 orientation, respectively (Figure 7c,d, highlighted with orange labels).

In addition to docking and generating MDs in the inactive‐state crystal structure of the μ opioid receptor, this process was carried out in the active‐state crystal structure (Huang et al., 2015). Docking and MDs again revealed two potential binding orientations for fentanyl and carfentanil (Figures S8 and S9). In these orientations (termed as pose3 and pose4), fentanyl and carfentanil in large matched the behaviour of the ligands modelled in the inactive‐state crystal structure.

## 4 DISCUSSION

In this study, we have investigated the signalling by fentanyls through the G protein and β‐arrestin 2 pathways using BRET assays, electrophysiological recordings of GIRK currents in rat LC neurones and an ELISA to determine agonist‐induced cell surface loss of μ opioid receptors. Calculations of bias using our signalling studies revealed that carfentanil is a β‐arrestin 2 biased agonist. This bias was substantial and could also be clearly seen when other signalling assays were analysed such as GIRK currents and cell surface receptor loss. We did not find the other fentanyls studied to display bias in any direction relative to the reference agonist DAMGO, unlike a previous study that reported fentanyl to be a β‐arrestin‐biased agonist (Schmid et al., 2017; see also Kelly, Sutcliffe, et al., 2023; Kelly, Conibear, et al., 2023). The β‐arrestin 2 bias of carfentanil was likely due to the high potency of carfentanil relative to DAMGO in the β‐arrestin 2 recruitment BRET assay compared with G protein‐dependent assays.

Whilst in ligand binding assays carfentanil has been reported to have a high affinity for the μ opioid receptor (Lipinski et al., 2019), it is less clear what the efficacy of carfentanil at the receptor is relative to other agonists such as DAMGO and fentanyl. We explored this in the present study by decreasing receptor reserve with the addition of the irreversible antagonist β‐FNA, which enables the relative efficacies of ligands to be determined. Such an approach has previously been employed by ourselves (Bailey et al., 2009; Rivero et al., 2012) and others (Singleton et al., 2021) to investigate the relative efficacy of agonists at the μ opioid receptor. The data from the present study revealed that DAMGO displays significantly higher efficacy for G protein activation than fentanyl or carfentanil. Whilst carfentanil has lower efficacy for G protein activation than DAMGO, it has higher efficacy for β‐arrestin 2 recruitment, which agrees with the conclusion that carfentanil is displaying β‐arrestin 2 bias relative to DAMGO. On the other hand, fentanyl displayed lower efficacy than DAMGO for G protein activation, and similar efficacy to DAMGO for β‐arrestin 2 recruitment. These findings are in agreement with previous studies for DAMGO and fentanyl where DAMGO was reported to exhibit higher efficacy for G protein activation than fentanyl at μ opioid receptors in cells in culture as well as in brain membranes (McPherson et al., 2010; Selley et al., 1998). Of interest, in the GIRK current assays in LC neurones, the E_max_ of DAMGO was significantly greater than that of carfentanil or fentanyl, possibly reflecting the higher efficacy of DAMGO for this G protein‐mediated response in this endogenous system. On the other hand, these differences in agonist E_max_ were not observed in the BRET cell signalling assays, which may reflect a higher receptor reserve and more signal amplification in the transfected cell system, and hence the presence of a tissue‐dependent maximum response. Variations in agonist E_max_ values for GPCR responses have been observed and discussed previously (Schrage et al., 2013; Schrage et al., 2016).

In LC neurones, carfentanil‐evoked GIRK currents were found to undergo desensitisation to a greater extent than those evoked by fentanyl, in spite of their apparent equivalent efficacy for GIRK channel activation in this system. This profile is in line with carfentanil's β‐arrestin 2‐biased signalling, such that the β‐arrestin‐dependent desensitisation in response to carfentanil would be more rapid and/or extensive than that induced by fentanyl (Lowe et al., 2015). This observation also reflects our previous findings for the β‐arrestin‐biased μ opioid receptor agonist endomorphin‐2 (McPherson et al., 2010), which was also found to produce a significantly greater degree of acute μ opioid receptor desensitisation than non‐biased opioids of equivalent G protein efficacy (Rivero et al., 2012).

From our molecular docking and MD simulations, we have determined two possible binding orientations for both fentanyl and carfentanil and in both the inactive‐state and active‐state crystal structures of the μ opioid receptor. Due to the stability of the opposing orientations and the flexibility of the ligands, we have not been able to determine which of these two orientations represents the “correct” physiological binding pose, if indeed there is just one. Determining the binding orientation of fentanyl and carfentanil has been a contentious issue, with a range of binding poses and orientations having been reported previously in the literature. These binding orientations can be grouped into either “phenethyl‐down” (Ellis et al., 2018; Eshleman et al., 2020; Lipinski et al., 2019; Lipiński et al., 2019; Subramanian et al., 2000), similar to our pose 1, or “phenethyl‐up” (de Waal et al., 2020; Ricarte et al., 2021; Tian et al., 2022), similar to our pose 2. In fact, some studies (in addition to the present one) have suggested both of these potential binding orientations (Dosen‐Micovic et al., 2006; Jaronczyk et al., 2017; Podlewska et al., 2020; Xie et al., 2022) and were also unable to elucidate the “correct” binding pose/orientation. It has also been suggested that the flexible nature of fentanyl may allow it to bind lower in the orthosteric side, potentially interacting with HIS297^6.52^ over ASP147^3.32^ (Mahinthichaichan et al., 2021; Vo et al., 2021), and we have also observed this pose in our MD studies (Ramos‐Gonzalez et al., unpublished observations). Very recently, three separate studies have reported cryo‐EM structures of fentanyl or fentanyl analogues bound to the μ opioid receptor (Faouzi et al., 2022; Qu et al., 2022; Zhuang et al., 2022); in each of these structures, the fentanyl ligand is oriented with its phenethyl towards a hydrophobic pocket between TM2/3 and its *N*‐phenyl‐propanamide towards TM5/6. These orientations of fentanyl are the same as those described herein and referred to as pose2 (Figure 7b) or pose4 (Figure S8b) in the inactive‐ and active‐state crystal structures, respectively. Thus, our reported poses2 and 4 appear to represent physiological ligand binding poses of fentanyl. Nevertheless, it should be noted that cryo‐EM provides a single snapshot and the computational evidence for more than one binding orientation of fentanyl remains an intriguing finding.

As we have shown that carfentanil is β‐arrestin‐biased whilst fentanyl does not display bias, we are interested in determining whether the interactions of carfentanil's additional 4‐carbomethoxy group may be involved in determining the β‐arrestin bias of this ligand. In our simulations, two residues have been highlighted as interacting with carfentanil's 4‐carbomethoxy grouping, depending on the orientation of the ligand in the orthosteric pocket, TYR128^2.64^ or ILE296^6.51^. The interaction between carfentanil's 4‐carbomethoxy group and ILE296^6.51^ has been observed in previous MD studies of carfentanil (de Waal et al., 2020), whilst other studies also highlight the interaction between carfentanil's 4‐carbomethoxy group and residues in TM6/7 (Lipiński et al., 2019; Xie et al., 2022). This residue, ILE296^6.51^, was also shown to be in proximity to both fentanyl and lofentanil in the recently published cryo‐EM structures (Faouzi et al., 2022; Qu et al., 2022; Zhuang et al., 2022). TYR128^2.64^ has not been previously highlighted as interacting with carfentanil's 4‐carbomethoxy group; however, it was highlighted as an important residue in the cryo‐EM structure of lofentanil in the μ opioid receptor as it forms a water mediated bridge with GLU124^2.60^ (Qu et al., 2022). We are currently investigating how mutating either of these residues to alanine may affect carfentanil's binding, signalling and degree of β‐arrestin bias.

Of potential interest, in a functional assay using naloxone as an antagonist, we find that the rate of dissociation of carfentanil from the μ opioid receptor is markedly slower than that of either DAMGO or fentanyl (Alhosan, Kelly and Henderson, unpublished findings/manuscript in preparation). A link between ligand binding kinetics and ligand bias has previously been suggested (Klein Herenbrink et al., 2016) whilst the agonist dissociation rate of ligands from the 5‐HT_2B_ receptor has previously been linked to β‐arrestin bias (Wacker et al., 2017).

The in vivo significance of carfentanil's β‐arrestin signalling bias described here remains to be fully explored but is a matter of some interest given carfentanil's high potential for toxicity in humans (George et al., 2010; Lust et al., 2011) along with increasing evidence of abuse of this drug (Leen & Juurlink, 2019). With carfentanil inducing a greater in vitro μ opioid receptor desensitisation relative to fentanyl, it is interesting to postulate whether carfentanil would produce a greater degree of tolerance than fentanyl in vivo (Dang & Christie, 2012; Kliewer et al., 2019, 2020; Whistler et al., 1999). However, the cellular mechanisms underlying the in vivo tolerance of even well studied μ opioid receptor agonists are complex and not fully understood (Groom et al., 2023; Jullié et al., 2022). Whilst detailed studies of the in vivo effects of carfentanil are limited, a recent study reported that the extent of carfentanil's abuse potential assessed using conditioned place preference, drug self‐administration and opioid withdrawal assays in mice was similar to that induced by fentanyl and heroin (Wei et al., 2023). Apart from carfentanil, as expected, being much more potent at inducing these behaviours than either fentanyl or heroin, the effects produced by the three agonists were essentially the same (Wei et al., 2023). The large variation in the ability of these three μ opioid receptor agonists to promote β‐arrestin recruitment to the receptor (Bohn et al., 2004; McPherson et al., 2010; the present study) suggests that β‐arrestin recruitment may not be important for some opioid agonist effects related to drug abuse, but instead and more likely G protein activation underlies these particular in vivo effects (see Kelly, Sutcliffe, et al., 2023). However, the measures used in the study by Wei et al. (2023) reflect relatively acute adaptations, and it is conceivable that β‐arrestins play a role in longer term adaptations occurring in drug abuse. On the other hand, the in vivo potency with which carfentanil can induce analgesia in mice (Suzuki & El‐Haddad, 2017) or positive reinforcing effects in monkeys (Gerak & France, 2023) relative to fentanyl is greater than can be explained by differences in receptor binding affinity or by relative potency in G protein cell signalling assays. Whether this difference is instead related in any way to carfentanil's β‐arrestin‐bias, or to other potential actions of this drug (e.g., Kelly, Sutcliffe, et al., 2023; Kelly, Conibear, et al., 2023; Sutcliffe et al., 2022), remains to be determined.

## 5 CONCLUSIONS

Our data reveal that carfentanil is a β‐arrestin‐biased agonist at the μ opioid receptor. Other fentanyls including fentanyl itself are not β‐arrestin‐biased. Whilst carfentanil does not have particularly high efficacy for G protein signalling at the μ opioid receptor, it does indeed have high efficacy for β‐arrestin 2 recruitment, and it is this combination of effects which is likely to underlie its β‐arrestin 2‐bias. It is possible that the β‐arrestin bias of carfentanil produces effects in vivo distinct from those of other fentanyls, perhaps related to altered receptor phosphorylation, trafficking or arrestin‐dependent signalling, or even more efficient uncoupling of G protein‐mediated responses contributing to tolerance. Further in vivo studies with carfentanil will be required to determine if this is the case.

### DECLARATION OF TRANSPARENCY AND SCIENTIFIC RIGOUR

This Declaration acknowledges that this paper adheres to the principles for transparent reporting and scientific rigour of preclinical research as stated in the *BJP* guidelines for Design and Analysis, and Animal Experimentation, and as recommended by funding agencies, publishers and other organisations engaged with supporting research.

## AUTHOR CONTRIBUTIONS

*Participated in research design*: Nokomis Ramos‐Gonzalez, Sam Groom, Chris P. Bailey, Richard B. Sessions, Graeme Henderson and Eamonn Kelly. *Conducted experiments*: Nokomis Ramos‐Gonzalez, Sam Groom, Katy J. Sutcliffe and Sukhvinder Bancroft. *Performed data analysis*: Nokomis Ramos‐Gonzalez, Sam Groom, Katy J. Sutcliffe and Sukhvinder Bancroft. *Contributed to the writing of the manuscript*: all authors.

## CONFLICT OF INTEREST STATEMENT

The authors declare no conflicts of interest.

## Supporting information


**FIGURE S1.** **Graphs showing ΔΔLog(τ/K**_**A**_ **) values**. (a) Graph showing ΔΔLog(τ/K_A_) values for the comparison between cell surface receptor loss and β‐arrestin 2 recruitment. (b) Graph showing ΔΔLog(τ/K_A_) values for the comparison between G protein activation BRET and GIRK current. DAMGO was used as the reference agonist in all comparisons. Bars are mean ± SEM, n = 5. A one‐way ANOVA with Dunnett's multiple comparisons post‐hoc test was used to determine statistical significance relative to DAMGO (p ≤ 0.1, *; p ≤ 0.01, **; p ≤ .001, ***).
**Figure S2. Log concentration‐response curves in the presence and absence of β‐FNA with equieffective lines drawn on.** Graphs show the log concentration response curves for DAMGO (a, b), fentanyl (c, d) and carfentanil (e, f) in the absence (colour) and presence (grey) of β‐FNA, for the G protein activation and β‐arrestin 2 BRET assays, as seen in Figure 4; datapoints are mean ±SEM, n = 5. On each graph parallel lines have been drawn between the two curves joining points where the level of agonist response is the same (i.e. points on the curves where the absolute response to the stimulus is the same) in the absence (termed [A]) and presence (termed [A']) of β‐FNA. This was then used to create a plot of [A] vs. [A'] for each agonist, in each assay (see Figure S3).
**Figure S3. Plot of [A] versus [A'] for G protein activation and β‐arrestin 2 recruitment BRET assays for DAMGO, fentanyl and carfentanil.** In each graph [A] vs. [A'], which was calculated as shown in Figure S2, has been plotted for DAMGO (a, b), fentanyl (c, d) and carfentanil (e, f), for the G protein activation and β‐arrestin 2 recruitment BRET. The data were then fitted to Equation 2 in the Methods to give K_A_ and q_functional_ values for each agonist in both assays. The values of [A] and K_A_ were then used in Equation 3 in the Methods to calculate occupancy for each drug, see Figure 5.
**Figure S4. Plot of response versus fractional receptor occupancy for G protein activation and β‐arrestin 2 recruitment BRET assays for DAMGO, fentanyl and carfentanil.** Graphs show response (data collected as shown in Figure 4) vs. occupancy (data calculated as described in figure 5, and Methods section 2.7). Data has been normalised to % of the maximum response for each drug. This is shown for DAMGO (a,b), fentanyl (c, d) and carfentanil (e, f), for both G protein activation (a,c,e) and β‐arrestin 2 recruitment (b,d,f). These graphs were used to calculate the % occupancy required to give 50% of the maximal response for each drug in each assay (see Table 2).
**Figure S5. Lack of effect of Compound 101 on the size of the peak carfentanil‐activated GIRK current in rat LC neurones**. Graph showing the peak GIRK currents evoked by 1 μM DAMGO alone, 1 μM fentanyl alone, and 100 nM carfentanil in the presence or absence of 30 μM Compound 101 (CPD101). The magnitude of GIRK currents is normalised to the maximal α2‐adrenoceptor‐mediated current evoked by noradrenaline (NA; 100 μM) in the same cell. There was no significant difference between the peak GIRK current evoked by carfentanil in the presence or absence of Compound 101 (p = 0.33). Error bars show ± SEM, where n = 5, except for fentanyl where n = 4.
**Figure S6. Traces showing the ligand RMSD and proximity to ASP147** ^**3.32**^ **for 1 μs MDs of fentanyl and carfentanil in the inactive‐state μ opioid receptor structure.** The data relate to the poses shown in figure 7 and Figure S7. Ligand RMSD is shown in colour for fentanyl pose1 (a, blue), fentanyl pose2 (b, blue), carfentanil pose1 (c, green) and carfentanil pose2 (d, green), RMSD is calculated relative to the first frame of the simulation. The distance between the ligand protonated nitrogen and conserved residue ASP147^3.32^ is shown on each plot in grey.
**Figure S7. Images of fentanyl and carfentanil in the inactive‐state μ opioid receptor after 1 μs MD.** Images are shown from the side of the receptor, TMDs have been labelled and TM7 has been removed for clarity. (a) fentanyl pose1, phenethyl towards intracellular side of receptor; (b) fentanyl pose 2, phenethyl towards extracellular side of receptor; (c) carfentanil pose 1, phenethyl towards intracellular side of receptor; (d) carfentanil pose 2, phenethyl towards extracellular side of receptor. Conserved residue ASP147^3.32^ (D147) is shown in purple. Oxygen atoms are shown in red, nitrogen atoms are shown in blue.
**Figure S8. Traces showing the ligand RMSD and proximity to ASP147** ^**3.32**^ **for 1 μs MDs of fentanyl and carfentanil in the active‐state μ opioid receptor structure.** Ligand RMSD is shown in colour for fentanyl pose3 (a, dark blue), fentanyl pose4 (b, dark blue), carfentanil pose3 (c, dark green) and carfentanil pose4 (d, dark green), RMSD is calculated relative to the first frame of the simulation. The distance between the ligand protonated nitrogen and conserved residue ASP147^3.32^ is shown on each plot in grey.
**Figure S9. Images of fentanyl and carfentanil in the active‐state μ opioid receptor after 1 μs MD.** Images are shown from the side of the receptor, TMDs have been labelled and TM7 has been removed for clarity. Images are of the final frame of 1 μs MDs of fentanyl and carfentanil in the active‐state receptor (PDB: 5C1M). (a) Fentanyl pose3, phenethyl towards intracellular side of receptor; (b) fentanyl pose 4, phenethyl towards extracellular side of receptor; (c) carfentanil pose 3, phenethyl towards intracellular side of receptor; (d) carfentanil pose 4, phenethyl towards extracellular side of receptor. Conserved residue ASP147^3.32^ (D147) is shown in purple. Oxygen atoms are shown in red, nitrogen atoms are shown in blue.
**Table S1. Table showing the raw values from the bias calculations.** In the table Log(τ/K_A_) values are shown for each agonist in each of the four assays, these values were calculated from fitting the data to the operational model of pharmacological agonism (see section 2.6 in Methods). ΔLog(τ/K_A_) is shown for each agonist, using DAMGO as the reference agonist. ΔΔLog(τ/K_A_) values are also shown for each agonist, for each pathway comparison. Values are means ± SEM. Grey boxes indicate where an agonist was not included in that particular assay.
**Table S2. Table showing the residues of the μ opioid receptor that were within a 4 Å proximity to fentanyl and carfentanil during the 1 μs MDs in the inactive state μ opioid receptor.** Residues within 4 Å of fentanyl and carfentanil during the 1 μs MDs were calculated for both pose1 and pose2 for the two ligands. The numbers show the % of the MDs that the residue was within 4 Å. The part of the receptor where each residue is found is also indicated. Only residues that were within 4 Å for more than 50% of the MDs time period is shown in order to highlight residues that may be forming stable interactions with the ligands. ILE296^6.51^ has been included for carfentanil Pose2 as, though it is within 4 Å of carfentanil for less than 50% of the simulation, it is still a potentially key interaction with carfentanil's 4‐carbomethoxy group.

## Data Availability

All relevant data are included in the figures and tables. Additional primary data sources supporting the study are available upon reasonable request from the corresponding author.

## References

[bph16084-bib-0001] Alexander, S. P. , Christopoulos, A. , Davenport, A. P. , Kelly, E. , Mathie, A. , Peters, J. A. , Veale, E. L. , Armstrong, J. F. , Faccenda, E. , Harding, S. D. , Pawson, A. J. , Southan, C. , Davies, J. A. , Abbracchio, M. P. , Alexander, W. , Al‐hosaini, K. , Bäck, M. , Barnes, N. M. , Bathgate, R. , … Ye, R. D. (2021). The concise guide to pharmacology 2021/22: G protein‐coupled receptors. British Journal of Pharmacology, 178(S1), S27–S156. 10.1111/bph.15538 34529832

[bph16084-bib-0002] Al‐Hasani, R. , & Bruchas, M. R. (2011). Molecular mechanisms of opioid receptor‐dependent signaling and behaviour. Anesthesiology, 115(6), 1363–1381. 10.1097/ALN.0b013e318238bba6 22020140 PMC3698859

[bph16084-bib-0003] Bailey, C. P. , Couch, D. , Johnson, E. , Griffiths, K. , Kelly, E. , & Henderson, G. (2003). Mu‐opioid receptor desensitization in mature rat neurons: Lack of interaction between DAMGO and morphine. The Journal of Neuroscience, 23, 10515–10520. 10.1523/JNEUROSCI.23-33-10515.2003 14627635 PMC6740932

[bph16084-bib-0004] Bailey, C. P. , Llorente, J. , Gabra, B. H. , Smith, F. L. , Dewey, W. L. , Kelly, E. , & Henderson, G. (2009). Role of protein kinase C and μ‐opioid receptor (MOPr) desensitization in tolerance to morphine in rat locus coeruleus neurons. European Journal of Neuroscience, 29, 307–318. 10.1111/j.1460-9568.2008.06573.x 19200236 PMC2695152

[bph16084-bib-0005] Ballesteros, J. A. , & Weinstein, H. (1995). Integrated methods for the construction of three‐dimensional models and computational probing of structure‐function relations in G protein‐coupled receptors. Methods in Neuroscience, 25, 366–428. 10.1016/S1043-9471(05)80049-7

[bph16084-bib-0006] Black, J. W. , & Leff, P. (1983). Operational models of pharmacological agonism. Proceedings of the Royal Society of London ‐ Series B: Biological Sciences, 220(1219), 141–162. 6141562 10.1098/rspb.1983.0093

[bph16084-bib-0007] Bohn, L. M. , Dykstra, L. A. , Lefkowitz, R. J. , Caron, M. G. , & Barak, L. S. (2004). Relative opioid efficacy is determined by the complements of the G protein‐coupled receptor desensitization machinery. Molecular Pharmacology, 66(1), 106–112. 10.1124/mol.66.1.106 15213301

[bph16084-bib-0008] Burns, S. M. , Cunningham, C. W. , & Mercer, S. L. (2018). DARK classics in chemical neuroscience: Fentanyl. ACS Chemical Neuroscience, 9, 2428–2437. 10.1021/acschemneuro.8b00174 29894151

[bph16084-bib-0009] CDC & National Center for Health Statistics . (2022). U.S. overdose deaths in 2021 increased half as much as in 2020—But are still up 15%.

[bph16084-bib-0010] Ciccarone, D. (2017). Fentanyl in the US heroin supply: A rapidly changing risk environment. International Journal of Drug Policy, 46, 107–111. 10.1016/j.drugpo.2017.06.010 28735776 PMC5742018

[bph16084-bib-0011] Comer, S. D. , & Cahill, C. M. (2019). Fentanyl: Receptor pharmacology, abuse potential, and implications for treatment. Neuroscience and Biobehavioral Reviews, 106, 49–57. 10.1016/j.neubiorev.2018.12.005 30528374 PMC7233332

[bph16084-bib-0012] Curtis, M. J. , Alexander, S. P. H. , Cirino, G. , George, C. H. , Kendall, D. A. , Insel, P. A. , Izzo, A. A. , Ji, Y. , Panettieri, R. A. , Patel, H. H. , Sobey, C. G. , Stanford, S. C. , Stanley, P. , Stefanska, B. , Stephens, G. J. , Teixeira, M. M. , Vergnolle, N. , & Ahluwalia, A. (2022). Planning experiments: Updated guidance on experimental design and analysis and their reporting III. British Journal of Pharmacology, 179, 3907–3913. 10.1111/bph.15868 35673806

[bph16084-bib-0013] Dang, V. C. , & Christie, M. J. (2012). Mechanisms of rapid opioid receptor desensitization, resensitization and tolerance in brain neurons. British Journal of Pharmacology, 165, 1704–1716. 10.1111/j.1476-5381.2011.01482.x 21564086 PMC3372824

[bph16084-bib-0014] de Waal, P. W. , Shi, J. , You, E. , Wang, X. , Melcher, K. , Jiang, Y. , Xu, H. E. , & Dickson, B. M. (2020). Molecular mechanisms of fentanyl mediated β‐arrestin biased signalling. PLOS Computational Biology, 16(4):e1007394. 10.1371/journal.pcbi.1007394 32275713 PMC7176292

[bph16084-bib-0015] Denis, C. , Saulière, A. , Galandrin, S. , Sénard, J. M. , & Galés, C. (2012). Probing heterotrimeric G protein activation: Applications to biased ligands. Current Pharmaceutical Design, 18, 128–144. 10.2174/138161212799040466 22229559 PMC3389521

[bph16084-bib-0016] Dennis, D. M. , Jacobson, K. , & Belardinelli, L. (1992). Evidence of spare A1‐adenosine receptors in guinea pig atrioventricular node. The American Journal of Physiology, 262, 661–671. 10.1152/ajpheart.1992.262.3.H661 PMC7001121 1558173

[bph16084-bib-0017] Dickson, C. J. , Madej, B. D. , Skjevik, A. A. , Betz, R. M. , Teigen, K. , Gould, I. R. , & Walker, R. C. (2014). Lipid14: The Amber lipid force field. Journal of Chemical Theory and Computation, 10(2), 865–879. 10.1021/ct4010307 24803855 PMC3985482

[bph16084-bib-0018] Dosen‐Micovic, L. , Ivanovic, M. , & Micovic, V. (2006). Steric interactions and the activity of fentanyl analogs at the μ‐opioid receptor. Bioorganic & Medicinal Chemistry, 14(9), 2887–2895. 10.1016/j.bmc.2005.12.010 16376082

[bph16084-bib-0019] Ellis, C. R. , Kruhlak, N. L. , Kim, M. T. , Hawkins, E. G. , & Stavitskaya, L. (2018). Predicting opioid receptor binding affinity of pharmacologically unclassified designer substances using molecular docking. PLoS ONE, 13(5), e0197734. 10.1371/journal.pone.0197734 29795628 PMC5967713

[bph16084-bib-0020] Eshleman, A. J. , Nagarajan, S. , Wolfrum, K. M. , Reed, J. F. , Nilsen, A. , Torralva, R. , & Janowsky, A. (2020). Affinity, potency, efficacy, selectivity, and molecular modeling of substituted fentanyls at opioid receptors. Biochemical Pharmacology, 182, 114293. 10.1016/j.bcp.2020.114293 33091380

[bph16084-bib-0021] Faouzi, A. , Wang, H. , Zaidi, S. A. , DiBerto, J. F. , Che, T. , Qu, Q. , Robertson, M. J. , Madasu, M. K. , el Daibani, A. , Varga, B. R. , Zhang, T. , Ruiz, C. , Liu, S. , Xu, J. , Appourchaux, K. , Slocum, S. T. , Eans, S. O. , Cameron, M. D. , al‐Hasani, R. , … Majumdar, S. (2022). Structure‐based design of bitopic ligands for the μ‐opioid receptor. Nature, 613, 767–774. 10.1038/s41586-022-05588-y 36450356 PMC10328120

[bph16084-bib-0022] Furchgott, R. F. (1966). The use of β‐haloalkylamines in the differentiation of receptors and in the determination of dissociation constants of receptor agonist complexes. In N. J. Harper & A. B. Simmonds (Eds.), Advances in drug research (Vol. 3) (pp. 21–55). Academic Press.

[bph16084-bib-0023] George, A. V. , Lu, J. J. , Pisano, M. V. , Metz, J. , & Erickson, T. B. (2010). Carfentanil—An ultra potent opioid. The American Journal of Emergency Medicine, 28(4), 530–532. 10.1016/j.ajem.2010.03.003 20466249

[bph16084-bib-0024] Gerak, L. R. , & France, C. P. (2023). Attenuation of the positive‐reinforcing effects of ultra‐potent fentanyl analogs, along with those of fentanyl and heroin, during daily treatment with methocinnamox (MCAM) in rhesus monkeys. The Journal of Pharmacology and Experimental Therapeutics, 384(3), 363–371. 36575032 10.1124/jpet.122.001267 PMC9976789

[bph16084-bib-0025] Groom, S. , Blum, N. K. , Conibear, A. E. , Disney, A. , Hill, R. , Husbands, S. M. , Li, Y. , Toll, L. , Kliewer, A. , Schulz, S. , & Henderson, G. (2023). A novel G protein‐biased agonist at the μ opioid receptor induces substantial receptor desensitisation through G protein‐coupled receptor kinase. British Journal of Pharmacology, 180(7), 943–957. 33245558 10.1111/bph.15334

[bph16084-bib-0026] Hill, R. , Santhakumar, R. , Dewey, W. , Kelly, E. , & Henderson, G. (2020). Fentanyl depression of respiration: Comparison with heroin and morphine. British Journal of Pharmacology, 177, 254–266. 10.1111/bph.14860 31499594 PMC6989952

[bph16084-bib-0027] Huang, W. , Manglik, A. , Venkatakrishnan, A. J. , Laeremans, T. , Feinberg, E. N. , Sanborn, A. L. , Kato, H. E. , Livingston, K. E. , Thorsen, T. S. , Kling, R. C. , Granier, S. , Gmeiner, P. , Husbands, S. M. , Traynor, J. R. , Weis, W. I. , Steyaert, J. , Dror, R. O. , & Kobilka, B. K. (2015). Structural insights into μ‐opioid receptor activation. Nature, 524(7565), 315–321. 10.1038/nature14886 26245379 PMC4639397

[bph16084-bib-0028] Jannetto, P. J. , Helander, A. , Garg, U. , Janis, G. C. , Goldberger, B. , & Ketha, H. (2019). The fentanyl epidemic and evolution of fentanyl analogs in the United States and the European Union. Clinical Chemistry, 65(2), 242–253. 10.1373/clinchem.2017.281626 30305277

[bph16084-bib-0029] Jaronczyk, M. , Lipinski, P. F. J. , Dobrowolski, J. C. , & Sadlej, J. (2017). The FMO analysis of the molecular interaction of fentanyl derivatives with the μ‐opioid receptor. Chemical Papers, 71, 1429–1443. 10.1007/s11696-017-0136-5

[bph16084-bib-0030] Jo, S. , Kim, T. , Iyer, V. G. , & Im, W. (2008). CHARMM‐GUI: A web‐based graphical user interface for CHARMM. Journal of Computational Chemistry, 29(11), 1859–1865. 10.1002/jcc.20945 18351591

[bph16084-bib-0031] Johnson, E. A. , Oldfield, S. , Braksator, E. , Gonzalez‐Cuello, A. , Couch, D. , Hall, K. J. , Mundell, S. J. , Bailey, C. P. , Kelly, E. , & Henderson, G. (2006). Agonist‐selective mechanisms of μ‐opioid receptor desensitization in human embryonic kidney 293 cells. Molecular Pharmacology, 70, 676–685. 10.1124/mol.106.022376 16682505

[bph16084-bib-0032] Jorgensen, W. L. , Chandrasekhar, J. , Madura, J. D. , Impey, R. W. , & Klein, M. L. (1983). Comparison of simple potential functions for simulating liquid water. The Journal of Chemical Physics, 79(2), 926–935. 10.1063/1.445869

[bph16084-bib-0033] Jullié, D. , Benitez, C. , Knight, T. A. , Simic, M. S. , & von Zastrow, M. (2022). Endocytic trafficking determines cellular tolerance of presynaptic opioid signaling. eLife, 11(e81298). 10.7554/eLife.81298 PMC9708073 36377786

[bph16084-bib-0034] Keith, D. E. , Murray, S. R. , Zaki, P. A. , Chu, P. C. , Lissin, D. V. , Kang, L. , Evans, C. J. , & von Zastrow, M. (1996). Morphine activates opioid receptors without causing their rapid internalization. Journal of Biological Chemistry, 271(32), 19021–19024. 10.1074/jbc.271.32.19021 8702570

[bph16084-bib-0035] Kelly, E. (2013). Efficacy and ligand bias at the μ‐opioid receptor. British Journal of Pharmacology, 169, 1430–1446. 10.1111/bph.12222 23646826 PMC3724102

[bph16084-bib-0036] Kelly, E. , Bailey, C. P. , & Henderson, G. (2008). Agonist‐selective mechanisms of GPCR desensitization. British Journal of Pharmacology, 153, 379–388. 10.1038/sj.bjp.0707604 PMC2268061 18059321

[bph16084-bib-0093] Kelly, E. , Conibear, A. , & Henderson, G. (2023). Biased agonism: Lessons from studies of opioid receptor agonists. Annual Review of Pharmacology & Toxicology, 63, 491–515. 10.1146/annurev-pharmtox-052120-091058 36170657

[bph16084-bib-0037] Kelly, E. , Sutcliffe, K. , Cavallo, D. , Ramos‐Gonzalez, N. , Alhosan, N. , & Henderson, G. (2023). The anomalous pharmacology of fentanyl. British Journal of Pharmacology, 180(7), 797–812. 34030211 10.1111/bph.15573

[bph16084-bib-0038] Kenakin, T. , Watson, C. , Muniz‐Medina, V. , Christopoulos, A. , & Novick, S. (2012). A simple method for quantifying functional selectivity and agonist bias. ACS Chemical Neuroscience, 3(3), 193–203. 10.1021/cn200111m 22860188 PMC3369801

[bph16084-bib-0039] Kissin, I. (2010). The development of new analgesics over the past 50 years: A lack of real breakthrough drugs. Anesthesia and Analgesia, 110(3), 780–789. 10.1213/ANE.0b013e3181cde882 20185657

[bph16084-bib-0040] Klein Herenbrink, C. , Sykes, D. A. , Donthamsetti, P. , Canals, M. , Coudrat, T. , Shonberg, J. , Scammells, P. J. , Capuano, B. , Sexton, P. M. , Charlton, S. J. , Javitch, J. A. , Christopoulos, A. , & Lane, J. R. (2016). The role of kinetic context in apparent biased agonism at GPCRs. Nature Communications, 7(10842), 10842. 10.1038/ncomms10842 PMC4770093 26905976

[bph16084-bib-0041] Kliewer, A. , Gillis, A. , Hill, R. , Schmiedel, F. , Bailey, C. , Kelly, E. , Henderson, G. , Christie, M. J. , & Schulz, S. (2020). Morphine‐induced respiratory depression is independent of β‐arrestin 2 signalling. British Journal of Pharmacology, 177, 2923–2931. 10.1111/bph.15004 32052419 PMC7280004

[bph16084-bib-0042] Kliewer, A. , Schmiedel, F. , Sianati, S. , Bailey, A. , Bateman, J. T. , Levitt, E. S. , Williams, J. T. , Christie, M. J. , & Schulz, S. (2019). Phosphorylation‐deficient G‐protein‐biased μ opioid receptors improve analgesia and diminish tolerance but worsen opioid side effects. Nature Communications, 10(367), 367. 10.1038/s41467-018-08162-1 PMC6341117 30664663

[bph16084-bib-0043] Kolb, P. , Kenakin, T. , Alexander, S. P. H. , Bermudez, M. , Bohn, L. M. , Breinholt, C. S. , Bouvier, M. , Hill, S. J. , Kostenis, E. , Martemyanov, K. A. , Neubig, R. R. , Onaran, H. O. , Rajagopal, S. , Roth, B. L. , Selent, J. , Shukla, A. K. , Sommer, M. E. , & Gloriam, D. E. (2022). Community guidelines for GPCR ligand bias: IUPHAR review 32. British Journal of Pharmacology, 179(14), 3651–3674. 10.1111/bph.15811 35106752 PMC7612872

[bph16084-bib-0044] Kuczynska, K. , Grzonkowski, P. , Kacprzak, L. , & Zawilska, J. B. (2018). Abuse of fentanyl: An emerging problem to face. Forensic Science International, 289, 207–214. 10.1016/j.forsciint.2018.05.042 29902699

[bph16084-bib-0045] Leen, J. L. S. , & Juurlink, D. N. (2019). Carfentanil: A narrative review of its pharmacology and public health concerns. Canadian Journal of Anaesthesia, 66(4), 414–421. 10.1007/s12630-019-01294-y 30666589

[bph16084-bib-0046] Li, J. G. , Chen, C. , Yin, J. , Rice, K. , Zhang, Y. , Matecka, D. , de Riel, J. K. , Desjarlais, R. L. , & Liu‐Chen, L. Y. (1999). ASP147 in the third transmembrane helix of the rat μ opioid receptor forms ion‐pairing with morphine and naltrexone. Life Sciences, 65(2), 175–185. 10.1016/S0024-3205(99)00234-9 10416823

[bph16084-bib-0047] Lilley, E. , Stanford, S. C. , Kendall, D. E. , Alexander, S. P. , Cirino, G. , Docherty, J. R. , George, C. H. , Insel, P. A. , Izzo, A. A. , Ji, Y. , Panettieri, R. A. , Sobey, C. G. , Stefanska, B. , Stephens, G. , Teixeira, M. , & Ahluwalia, A. (2020). ARRIVE 2.0 and the British Journal of Pharmacology: Updated guidance for 2020. British Journal of Pharmacology, 177(16), 3611–3616. https://bpspubs.onlinelibrary.wiley.com/doi/full/10.1111/bph.15178 32662875 10.1111/bph.15178 PMC7393193

[bph16084-bib-0049] Lipinski, P. F. J. , Kosson, P. , Matalinska, J. , Roszkowski, P. , Czarnocki, Z. , Jaronczyk, M. , Misicka, A. , Dobrowolski, J. C. , & Sadlej, J. (2019). Fentanyl family at the μ‐opioid receptor: Uniform assessment of binding and computational analysis. Molecules, 24, 740. 10.3390/molecules24040740 30791394 PMC6412969

[bph16084-bib-0048] Lipiński, P. F. J. , Jarończyk, M. , Dobrowolski, J. C. , & Sadlej, J. (2019). Molecular dynamics of fentanyl bound to μ‐opioid receptor. Journal of Molecular Modeling, 25, 144. 10.1007/s00894-019-3999-2 31053968

[bph16084-bib-0050] Lowe, J. D. , Sanderson, H. S. , Cooke, A. E. , Ostovar, M. , Tsisanova, E. , Withey, S. L. , Chavkin, C. , Husbands, S. M. , Kelly, E. , Henderson, G. , & Bailey, C. P. (2015). Role of G protein–coupled receptor kinases 2 and 3 in μ‐opioid receptor desensitization and internalization. Molecular Pharmacology, 88, 347–356. 10.1124/mol.115.098293 26013542 PMC4518089

[bph16084-bib-0051] Lust, E. B. , Barthold, C. , Malesker, M. A. , & Wichman, T. O. (2011). Human health hazards of veterinary medications: Information for emergency departments. The Journal of Emergency Medicine, 40(2), 198–207. 10.1016/j.jemermed.2009.09.026 20045604

[bph16084-bib-0052] Mahinthichaichan, P. , Vo, Q. N. , Ellis, C. R. , & Shen, J. (2021). Kinetics and mechanism of fentanyl dissociation from the μ‐opioid receptor. JACS, 1, 2208–2215. 10.1021/jacsau.1c00341 PMC8715493 34977892

[bph16084-bib-0053] Maier, J. A. , Martinez, C. , Kasavajhala, K. , Wickstrom, L. , Hauser, K. E. , & Simmerling, C. (2015). ff14SB: Improving the accuracy of protein side chain and backbone parameters from ff99SB. Journal of Chemical Theory and Computation, 11(8), 3696–3713. 10.1021/acs.jctc.5b00255 26574453 PMC4821407

[bph16084-bib-0054] Manglik, A. , Kruse, A. C. , Kobilka, T. S. , Thian, F. S. , Mathiesen, J. M. , Sunahara, R. K. , Pardo, L. , Weis, W. I. , Kobilka, B. K. , & Granier, S. (2012). Crystal structure of the μ‐opioid receptor bound to a morphinan antagonist. Nature, 485(7398), 321–326. 10.1038/nature10954 22437502 PMC3523197

[bph16084-bib-0055] Matthes, H. W. , Maldonado, R. , Simonin, F. , Valverde, O. , Slowe, S. , Kitchen, I. , Befort, K. , Dierich, A. , Le Meur, M. , Dollé, P. , & Tzavara, E. (1996). Loss of morphine‐induced analgesia, reward effect and withdrawal symptoms in mice lacking the μ‐opioid receptor gene. Nature, 383(6603), 819–823. 10.1038/383819a0 8893006

[bph16084-bib-0056] McIntosh‐Smith, S. , Price, J. , Sessions, R. B. , & Ibarra, A. A. (2015). High performance in silico virtual drug screening on many‐core processors. The International Journal of High Performance Computing Applications, 29(2), 119–134. 10.1177/1094342014528252 25972727 PMC4425459

[bph16084-bib-0057] McPherson, J. L. , Rivero, G. , Baptist, M. C. , Llorente, F. J. , Al‐Sabah, S. , Krasel, C. , Dewey, W. L. , Bailey, C. P. , Rosethorne, E. M. , Charlton, S. J. , & Henderson, G. (2010). μ‐Opioid receptors: Correlation of agonist efficacy for signalling with ability to activate internalization. Molecular Pharmaceutics, 78(4), 756–766. 10.1124/mol.110.066613 PMC2981392 20647394

[bph16084-bib-0058] Molinari, P. , Casella, I. , & Costa, T. (2008). Functional complementation of high‐efficiency resonance energy transfer: A new tool for the study of protein binding interactions in living cells. The Biochemical Journal, 409, 251–261. 10.1042/BJ20070803 17868039

[bph16084-bib-0059] Morey, T. E. , Belardinelli, L. , & Dennis, D. M. (1998). Validation of Furchgott's method to determine agonist‐dependent A1‐adenosine receptor reserve in guinea‐pig atrium. British Journal of Pharmacology, 123, 1425–1433. 10.1038/sj.bjp.0701747 9579739 PMC1565302

[bph16084-bib-0060] North, R. A. , & Williams, J. T. (1985). On the potassium conductance increased by opioids in rat locus coeruleus neurones. The Journal of Physiology, 364, 265–280. 10.1113/jphysiol.1985.sp015743 2411916 PMC1192968

[bph16084-bib-0061] Pathan, H. , & Williams, J. (2012). Basic opioid pharmacology: An update. British Journal of Pain, 6(1), 11–16. 10.1177/2049463712438493 26516461 PMC4590096

[bph16084-bib-0062] Peng, P. W. H. , & Sandler, A. N. (1999). A review of the use of fentanyl analgesia in the management of acute pain in adults. Anesthesiology, 90, 579–599. 10.1097/00000542-199902000-00034 9952166

[bph16084-bib-0063] Percie du Sert, N. , Hurst, V. , Ahluwalia, A. , Alam, S. , Avey, M. T. , Baker, M. , Browne, W. J. , Clark, A. , Cuthill, I. C. , Dirnagl, U. , Emerson, M. , Garner, P. , Holgate, S. T. , Howells, D. W. , Karp, N. A. , Lazic, S. E. , Lidster, K. , MacCallum, C. J. , Macleod, M. , … Würbel, H. (2020). The ARRIVE guidelines 2.0: Updated guidelines for reporting animal research. PLoS Biology, 18(7), e3000410. 10.1371/journal.pbio.3000410 32663219 PMC7360023

[bph16084-bib-0064] Pettersen, E. F. , Goddard, T. D. , Huang, C. C. , Couch, G. S. , Greenblatt, D. M. , Meng, E. C. , & Ferrin, T. E. (2004). UCSF chimera? A visualization system for exploratory research and analysis. Journal of Computational Chemistry, 25(13), 1605–1612. 10.1002/jcc.20084 15264254

[bph16084-bib-0065] Podlewska, S. , Bugno, R. , Kudla, L. , Bojarski, A. J. , & Przewlocki, R. (2020). Molecular modeling of μ opioid receptor ligands with various functional properties: PZM21, SR‐17018, morphine, and fentanyl—Simulated interaction patterns confronted with experimental data. Molecules, 25, 4636. 10.3390/molecules25204636 33053718 PMC7594085

[bph16084-bib-0066] Qu, Q. , Huang, W. , Aydin, D. , Paggi, J. M. , Seven, A. B. , Wang, H. , Chakraborty, S. , Che, T. , DiBerto, J. F. , Robertson, M. J. , & Inoue, A. (2022). Insights into distinct signaling profiles of the μOR activated by diverse agonists. Nature Chemical Biology, 19(4), 423–430. 36411392 10.1038/s41589-022-01208-y PMC11098091

[bph16084-bib-0067] Ricarte, A. , Dalton, J. A. R. , & Giraldo, J. (2021). Structural assessment of agonist efficacy in the μ‐opioid receptor: Morphine and fentanyl elicit different activation patterns. Journal of Chemical Information and Modeling, 61, 1251–1274. 10.1021/acs.jcim.0c00890 33448226

[bph16084-bib-0068] Rivero, G. , Llorente, J. , McPherson, J. , Cooke, A. , Mundell, S. J. , McArdle, C. A. , Rosethorne, E. M. , Charlton, S. J. , Krasel, C. , Bailey, C. P. , Henderson, G. , & Kelly, E. (2012). Endomorphin‐2: A biased agonist at the μ‐opioid receptor. Molecular Pharmacology, 82(2), 178–188. 10.1124/mol.112.078659 22553358 PMC3400840

[bph16084-bib-0069] Roe, D. R. , & Cheatham, T. E. 3rd. (2013). PTRAJ and CPPTRAJ: Software for processing and analysis of molecular dynamics trajectory data. Journal of Chemical Theory and Computation, 9(7), 3084–3095. 10.1021/ct400341p 26583988

[bph16084-bib-0070] Schmid, C. L. , Kennedy, N. M. , Ross, N. C. , Cameron, M. D. , Bannister, T. D. , & Bohn, L. M. (2017). Bias factor and therapeutic window correlate to predict safer opioid analgesics. Cell, 171, 1165–1175. 10.1016/j.cell.2017.10.035 29149605 PMC5731250

[bph16084-bib-0091] Schrage, R. , de Min, A. , Hochheiser, K. , Kostenis, E. , & Mohr, K. (2016). Superagonism at G protein‐coupled receptors and beyond. British Journal of Pharmacology, 173, 3018–3027. 10.1111/bph.13278 26276510 PMC5338155

[bph16084-bib-0092] Schrage, R. , Seemann, W. K. , Klöckner, J. , Dallanoce, C. , Racké, K. , Kostenis, E. , De Amici, M. , Holzgrabe, U. , & Mohr, K. (2013). Agonists with supraphysiological efficacy at the muscarinic M2 ACh receptor. British Journal of Pharmacology, 169, 357–370. 10.1111/bph.12003 23062057 PMC3651662

[bph16084-bib-0071] Selley, D. E. , Liu, Q. , & Childers, S. R. (1998). Signal transduction correlates of mu opioid agonist intrinsic efficacy: Receptor‐stimulated [^35^S] GTPγS binding in mMOR‐CHO cells and rat thalamus. The Journal of Pharmacology and Experimental Therapeutics, 285(2), 496–505. 9580589

[bph16084-bib-0072] Shenoy, S. K. , & Lefkowitz, R. J. (2011). β‐Arrestin‐mediated receptor trafficking and signal transduction. Trends in Pharmacological Sciences, 32(9), 521–533. 10.1016/j.tips.2011.05.002 21680031 PMC3159699

[bph16084-bib-0073] Singleton, S. , Baptista‐Hon, D. T. , Edelsten, E. , McCaughey, K. S. , Camplisson, E. , & Hales, T. G. (2021). TRV130 partial agonism and capacity to induce anti‐nociceptive tolerance revealed through reducing available μ‐opioid receptor number. British Journal of Pharmacology, 178(8), 1855–1868. 10.1111/bph.15409 33555037

[bph16084-bib-0074] Stanley, T. H. (2014). The fentanyl story. The Journal of Pain, 15(12), 1215–1226. 10.1016/j.jpain.2014.08.010 25441689

[bph16084-bib-0075] Subramanian, G. , Paterlini, M. G. , Portoghese, P. S. , & Ferguson, D. M. (2000). Molecular docking reveals a novel binding site model for fentanyl at the μ‐opioid receptor. Journal of Medicinal Chemistry, 43(3), 381–391. 10.1021/jm9903702 10669565

[bph16084-bib-0076] Surratt, C. K. , Johnson, P. S. , Moriwaki, A. , Seidleck, B. K. , Blaschak, C. J. , Wang, J. B. , & Uhl, G. R. (1994). Mu opiate receptor: Charged transmembrane domain amino acids are critical for agonist recognition and intrinsic activity. The Journal of Biological Chemistry, 269(32), 20548–20553. 10.1016/S0021-9258(17)32028-8 8051154

[bph16084-bib-0077] Sutcliffe, K. J. , Corey, R. A. , Alhosan, N. , Cavallo, D. , Groom, S. , Santiago, M. , Bailey, C. , Charlton, S. J. , Sessions, R. B. , Henderson, G. , & Kelly, E. (2022). Interaction with the lipid membrane influences fentanyl pharmacology. Advances in Drug and Alcohol Research, 2, 10280. 10.3389/adar.2022.10280 35909438 PMC7613138

[bph16084-bib-0078] Sutcliffe, K. J. , Henderson, G. , Kelly, E. , & Sessions, R. B. (2017). Drug binding poses relate structure with efficacy in the μ opioid receptor. Journal of Molecular Biology, 429(12), 1840–1851. 10.1016/j.jmb.2017.05.009 28502792 PMC5472181

[bph16084-bib-0079] Suzuki, J. , & El‐Haddad, S. (2017). A review: Fentanyl and non‐pharmaceutical fentanyls. Drug and Alcohol Dependence, 171, 107–116. 10.1016/j.drugalcdep.2016.11.033 28068563

[bph16084-bib-0080] Tian, X. , Zhang, J. , Wang, S. , Gao, H. , Sun, Y. , Liu, X. , Fu, W. , Tan, B. , & Su, R. (2022). Tyrosine 7.43 is important for μ‐opioid receptor downstream signaling pathways activated by fentanyl. Frontiers in Pharmacology, 13, 919325. 10.3389/fphar.2022.919325 36120357 PMC9478952

[bph16084-bib-0081] Vo, Q. N. , Mahinthichaichan, P. , Shen, J. , & Ellis, C. R. (2021). How μ‐opioid receptor recognizes fentanyl. Nature Communications, 12, 984. 10.1038/s41467-021-21262-9 PMC7881245 33579956

[bph16084-bib-0082] Wacker, D. , Wang, S. , McCorvy, J. D. , Betz, R. M. , Venkatakrishnan, A. J. , Levit, A. , Lansu, K. , Schools, Z. L. , Che, T. , Nichols, D. E. , Shoichet, B. K. , Dror, R. O. , & Roth, B. L. (2017). Crystal structure of an LSD‐bound human serotonin receptor. Cell, 168(3), 377–389. 10.1016/j.cell.2016.12.033 28129538 PMC5289311

[bph16084-bib-0083] Wang, J. , Wolf, R. M. , Caldwell, J. W. , Kollman, P. A. , & Case, D. A. (2004). Development and testing of a general amber force field. Journal of Computational Chemistry, 25(9), 1157–1174. 10.1002/jcc.20035 15116359

[bph16084-bib-0084] Wei, J. , Lai, M. , Li, F. , Chen, Y. , Li, X. , Qiu, Y. , Shen, H. , Xu, P. , & di, B. (2023). Assessment of abuse potential of carfentanil. Addiction Biology, 28(2), e13265. 10.1111/adb.13265 36692872

[bph16084-bib-0085] Whistler, J. L. , Chuang, H. H. , Chu, P. , Jan, L. Y. , & von Zastrow, M. (1999). Functional dissociation of μ opioid receptor signaling and endocytosis: Implications for the biology of opiate tolerance and addiction. Neuron, 23(4), 737–746. 10.1016/S0896-6273(01)80032-5 10482240

[bph16084-bib-0086] Williams, J. T. , Ingram, S. L. , Henderson, G. , Chavkin, C. , von Zastrow, M. , Schulz, S. , Koch, T. , Evans, C. J. , & Christie, M. D. J. (2013). Regulation of μ‐opioid receptors: Desensitization, phosphorylation, internalization, and tolerance. Pharmacological Reviews, 65(1), 223–254. 10.1124/pr.112.005942 23321159 PMC3565916

[bph16084-bib-0087] Xie, B. , Goldberg, A. , & Shi, L. (2022). A comprehensive evaluation of the potential binding poses of fentanyl and its analogs at the μ‐opioid receptor. Computational and Structural Biotechnology Journal, 20, 2309–2321. 10.1016/j.csbj.2022.05.013 35615021 PMC9123087

[bph16084-bib-0088] Zawilska, J. B. , Kuczyńska, K. , Kosmal, W. , Markiewicz, K. , & Adamowicz, P. (2021). Carfentanil—From an animal anesthetic to a deadly illicit drug. Forensic Science International, 320, 110715. 10.1016/j.forsciint.2021.110715 33581655

[bph16084-bib-0089] Zhuang, Y. , Wang, Y. , He, B. , He, X. , Zhou, X. E. , Guo, S. , Rao, Q. , Yang, J. , Liu, J. , Zhou, Q. , Wang, X. , Liu, M. , Liu, W. , Jiang, X. , Yang, D. , Jiang, H. , Shen, J. , Melcher, K. , Chen, H. , … Xu, H. E. (2022). Molecular recognition of morphine and fentanyl by the human m‐opioid receptor. Cell, 185, 4361–4375. 10.1016/j.cell.2022.09.041 36368306

