# Supplementary material for: Carfentanil is a β‐arrestin‐biased agonist at the μ opioid receptor
Source: Br J Pharmacol. 2023 May 18;180(18):2341–60. doi: 10.1111/bph.16084 (PMC10952505; doi:10.1111/bph.16084)
Supplement: Supplementary file 1 — FIGURE S1. Graphs showing ΔΔLog(τ/K A ) values. (a) Graph showing ΔΔLog(τ/KA) values for the comparison between cell surface receptor loss and β‐arrestin 2 recruitment. (b) Graph showing ΔΔLog(τ/KA) values for the comparison between G protein activation BRET and GIRK current. DAMGO was used as the reference agonist in all comparisons. Bars are mean ± SEM, n = 5. A one‐way ANOVA with Dunnett's multiple comparisons post‐hoc test was used to determine statistical significance relative to DAMGO (p ≤ 0.1, *; p ≤ 0.01, **; p ≤ .001, ***). Figure S2. Log concentration‐response curves in the presence and absence of β‐FNA with equieffective lines drawn on. Graphs show the log concentration response curves for DAMGO (a, b), fentanyl (c, d) and carfentanil (e, f) in the absence (colour) and presence (grey) of β‐FNA, for the G protein activation and β‐arrestin 2 BRET assays, as seen in Figure 4; datapoints are mean ±SEM, n = 5. On each graph parallel lines have been drawn between the two curves joining points where the level of agonist response is the same (i.e. points on the curves where the absolute response to the stimulus is the same) in the absence (termed [A]) and presence (termed [A']) of β‐FNA. This was then used to create a plot of [A] vs. [A'] for each agonist, in each assay (see Figure S3). Figure S3. Plot of [A] versus [A'] for G protein activation and β‐arrestin 2 recruitment BRET assays for DAMGO, fentanyl and carfentanil. In each graph [A] vs. [A'], which was calculated as shown in Figure S2, has been plotted for DAMGO (a, b), fentanyl (c, d) and carfentanil (e, f), for the G protein activation and β‐arrestin 2 recruitment BRET. The data were then fitted to Equation 2 in the Methods to give KA and qfunctional values for each agonist in both assays. The values of [A] and KA were then used in Equation 3 in the Methods to calculate occupancy for each drug, see Figure 5. Figure S4. Plot of response versus fractional receptor occupancy for G protein activation and β‐a [file BPH-180-2341-s001.docx]

**SUPPLEMENTAL DATA**

**FIGURE S1- Graphs showing ΔΔLog(τ/K_A_) values**. (a) Graph showing ΔΔLog(τ/K_A_) values for the comparison between cell surface receptor loss and β-arrestin 2 recruitment. (b) Graph showing ΔΔLog(τ/K_A_) values for the comparison between G protein activation BRET and GIRK current. DAMGO was used as the reference agonist in all comparisons. Bars are mean ± SEM, n=5. A one-way ANOVA with Dunnett’s multiple comparisons post-hoc test was used to determine statistical significance relative to DAMGO (p ≤ 0.1, *; p ≤ 0.01, **; p ≤ 0.001, ***).


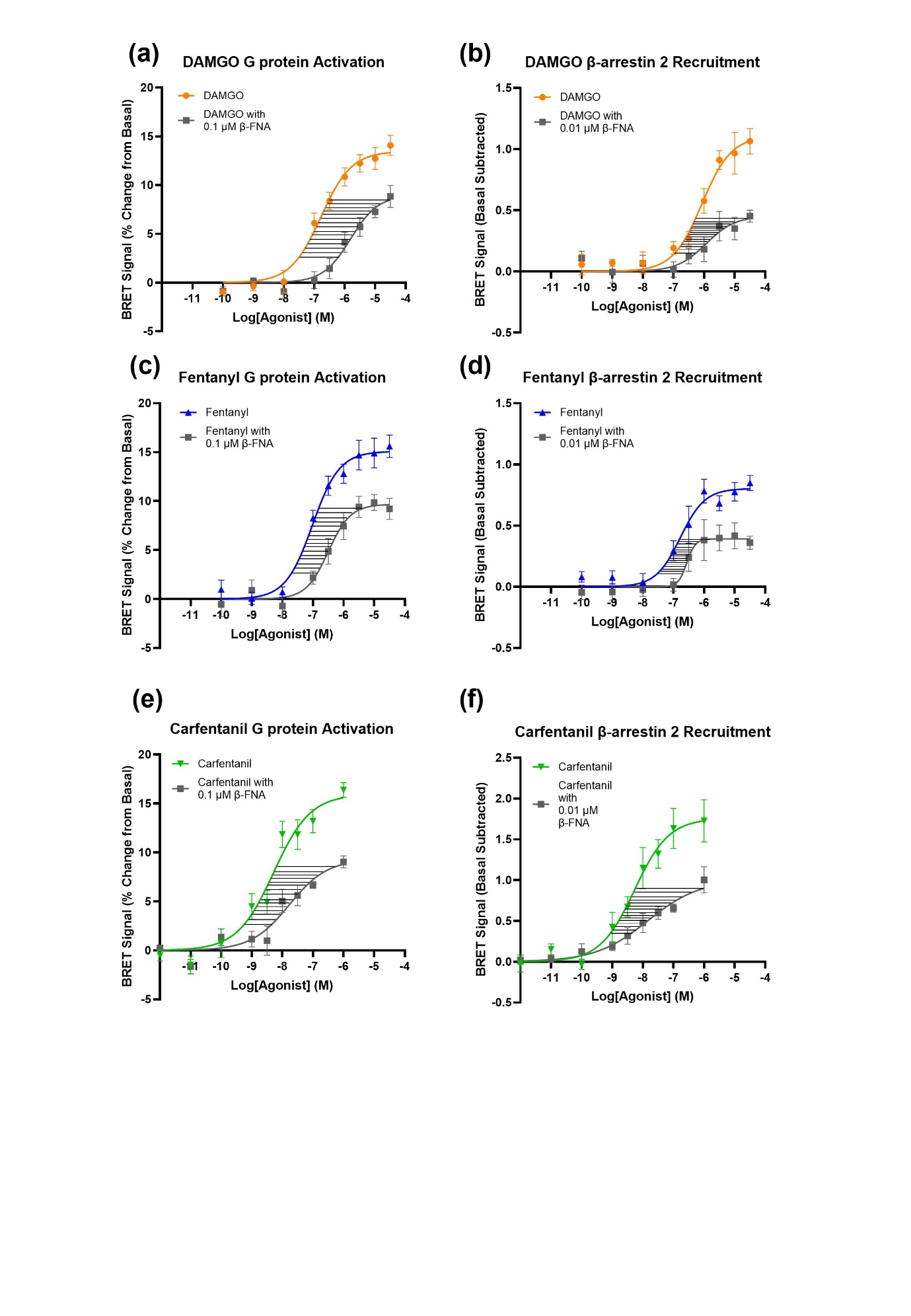


**FIGURE S2- Log concentration-response curves in the presence and absence of β-FNA with equieffective lines drawn on.** Graphs show the log concentration response curves for DAMGO (a, b), fentanyl (c, d) and carfentanil (e, f) in the absence (colour) and presence (grey) of β-FNA, for the G protein activation and β-arrestin 2 BRET assays, as seen in Figure 4; datapoints are mean ±SEM, n=5. On each graph parallel lines have been drawn between the two curves joining points where the level of agonist response is the same (i.e. points on the curves where the absolute response to the stimulus is the same) in the absence (termed [A]) and presence (termed [A’]) of β-FNA. This was then used to create a plot of [A] vs. [A’] for each agonist, in each assay (see Figure S3).


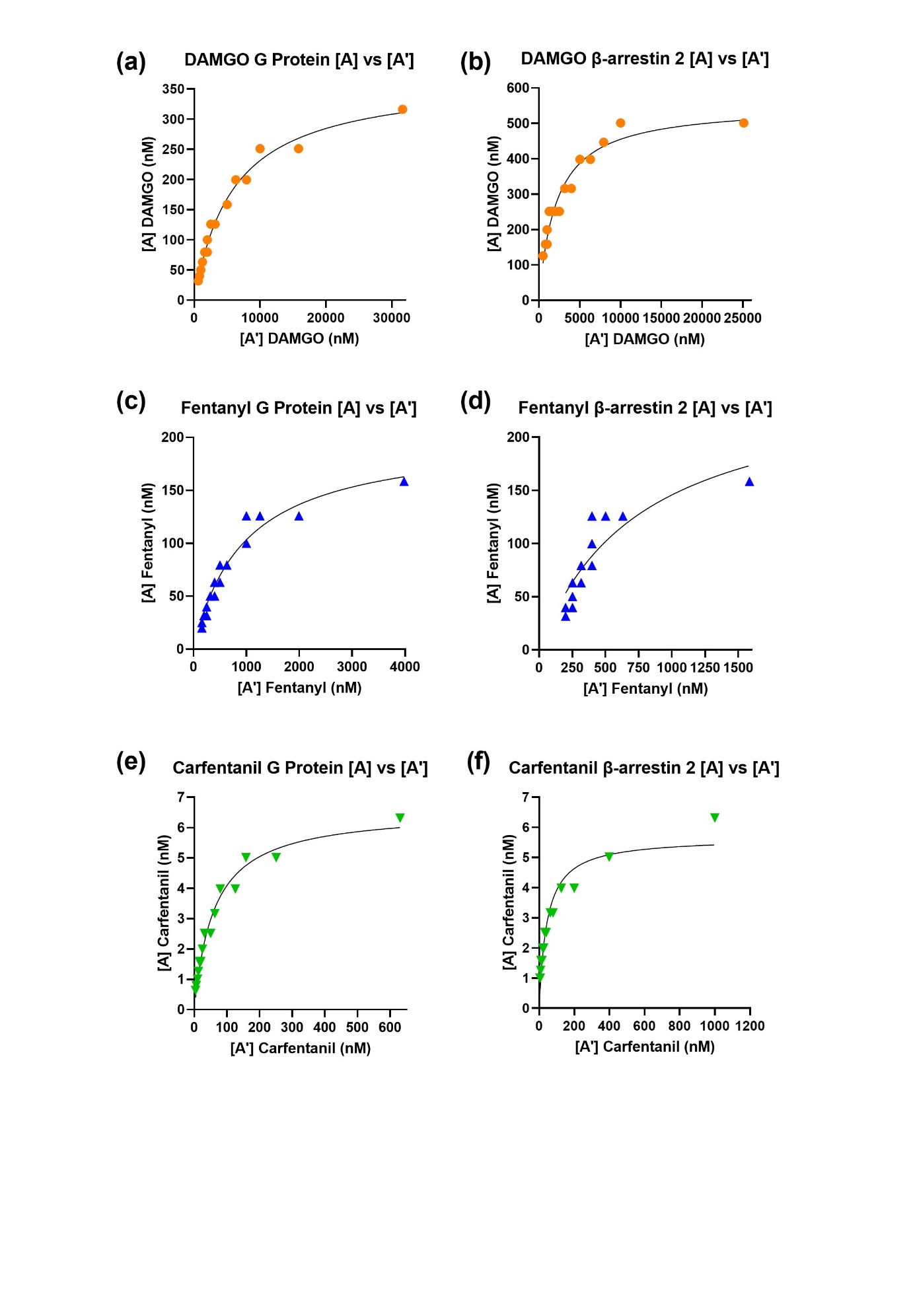


**FIGURE S3- Plot of [A] versus [A’] for G protein activation and β-arrestin 2 recruitment BRET assays for DAMGO, fentanyl and carfentanil.** In each graph [A] vs. [A’], which was calculated as shown in Figure S2, has been plotted for DAMGO (a, b), fentanyl (c, d) and carfentanil (e, f), for the G protein activation and β-arrestin 2 recruitment BRET. The data were then fitted to Equation 2 in the Methods to give K_A_ and q_functional_ values for each agonist in both assays. The values of [A] and K_A_ were then used in Equation 3 in the Methods to calculate occupancy for each drug, see Figure 5.


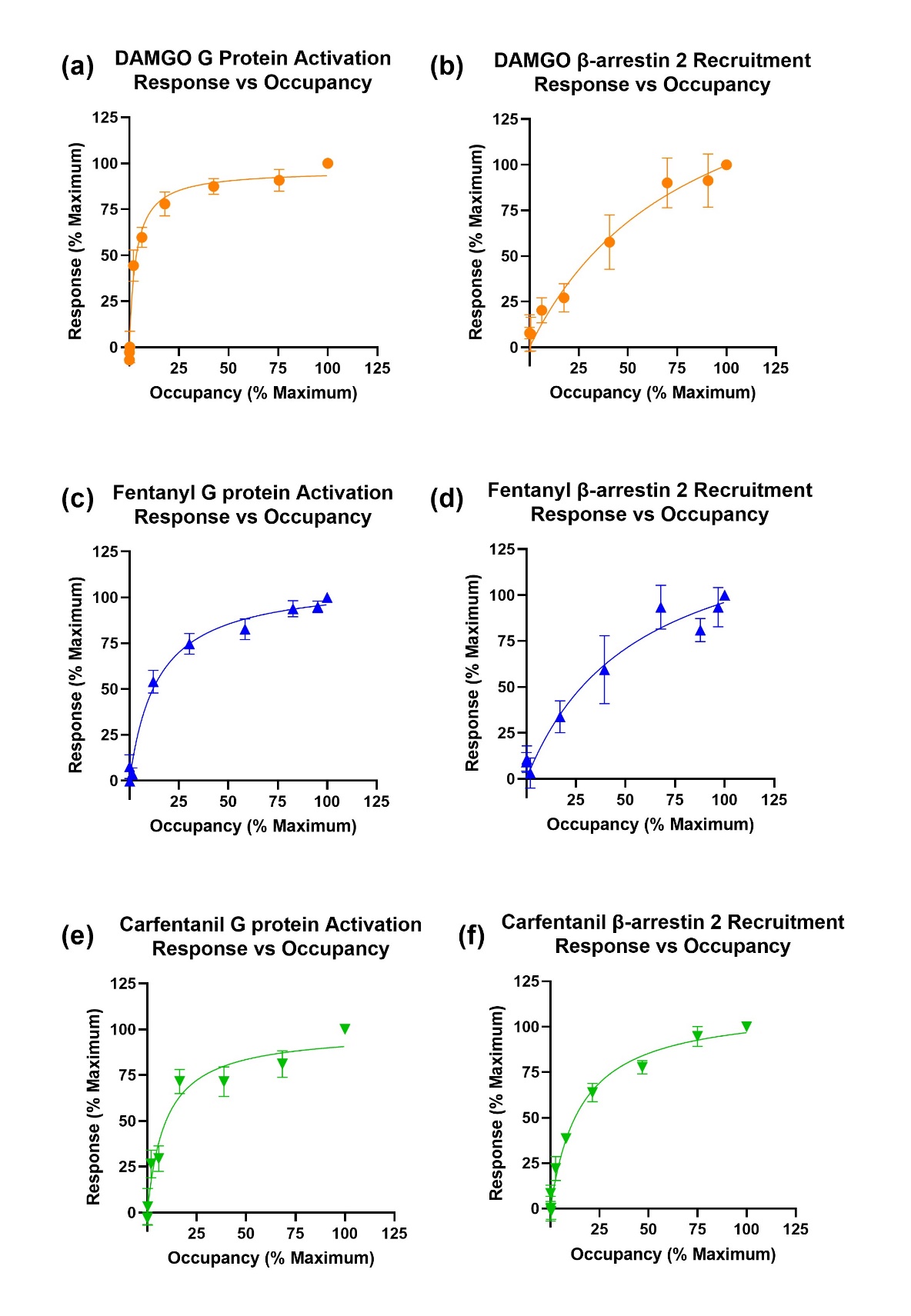


**FIGURE S4- Plot of response versus fractional receptor occupancy for G protein activation and β-arrestin 2 recruitment BRET assays for DAMGO, fentanyl and carfentanil.** Graphs show response (data collected as shown in Figure 4) vs. occupancy (data calculated as described in figure 5, and Methods section 2.7). Data has been normalised to % of the maximum response for each drug. This is shown for DAMGO (a ,b), fentanyl (c, d) and carfentanil (e, f), for both G protein activation (a,c,e) and β-arrestin 2 recruitment (b,d,f). These graphs were used to calculate the % occupancy required to give 50% of the maximal response for each drug in each assay (see Table 2).


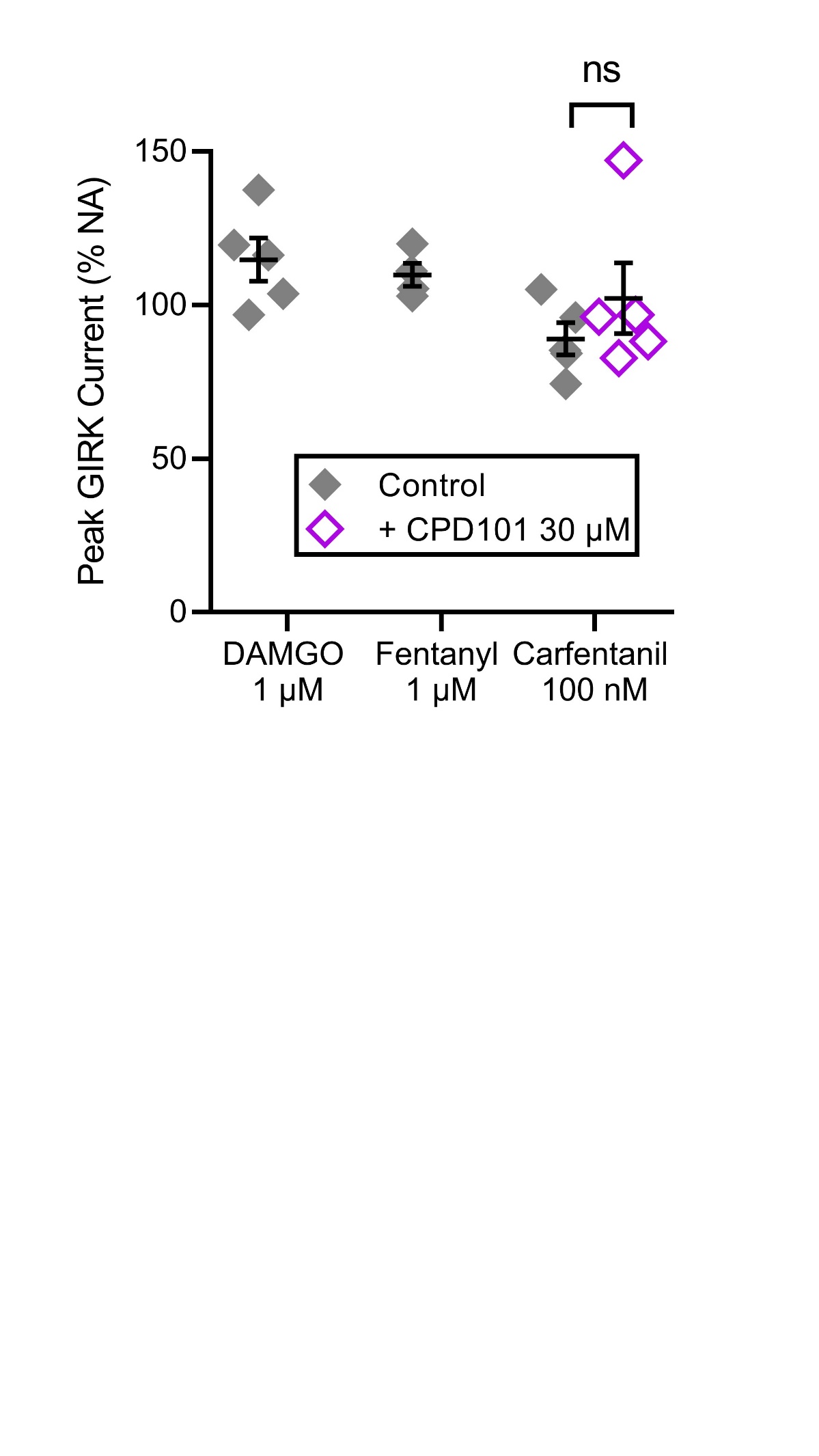


**FIGURE S5 – Lack of effect of CPD101 on the size of the peak carfentanil-activated GIRK current in rat LC neurones**. Graph showing the peak GIRK currents evoked by 1 µM DAMGO alone, 1 µM fentanyl alone, and 100 nM carfentanil in the presence or absence of 30 µM Compound 101 (CPD101). The magnitude of GIRK currents is normalised to the maximal α2-adrenoceptor-mediated current evoked by noradrenaline (NA; 100 μM) in the same cell. There was no significant difference between the peak GIRK current evoked by carfentanil in the presence or absence of Compound 101 (p=0.33). Error bars show ± SEM, where n=5, except for fentanyl where n=4.


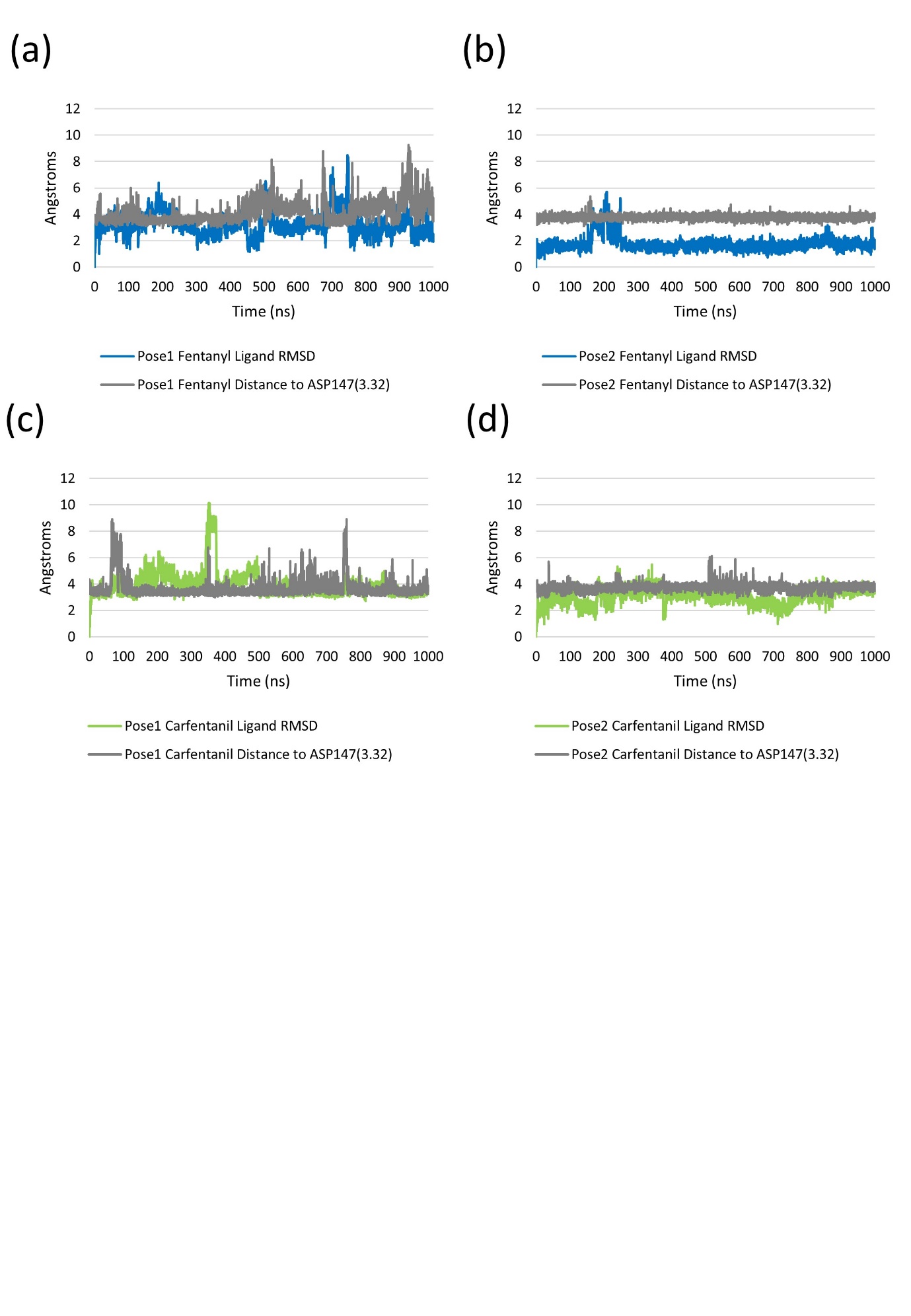


**FIGURE S6- Traces showing the ligand RMSD and proximity to ASP147^3.32^ for 1 µs MDs of fentanyl and carfentanil in the inactive-state µ opioid receptor structure.** The data relate to the poses shown in figure 7 and Figure S7. Ligand RMSD is shown in colour for fentanyl pose1 (a, blue), fentanyl pose2 (b, blue), carfentanil pose1 (c, green) and carfentanil pose2 (d, green), RMSD is calculated relative to the first frame of the simulation. The distance between the ligand protonated nitrogen and conserved residue ASP147^3.32^ is shown on each plot in grey.

**
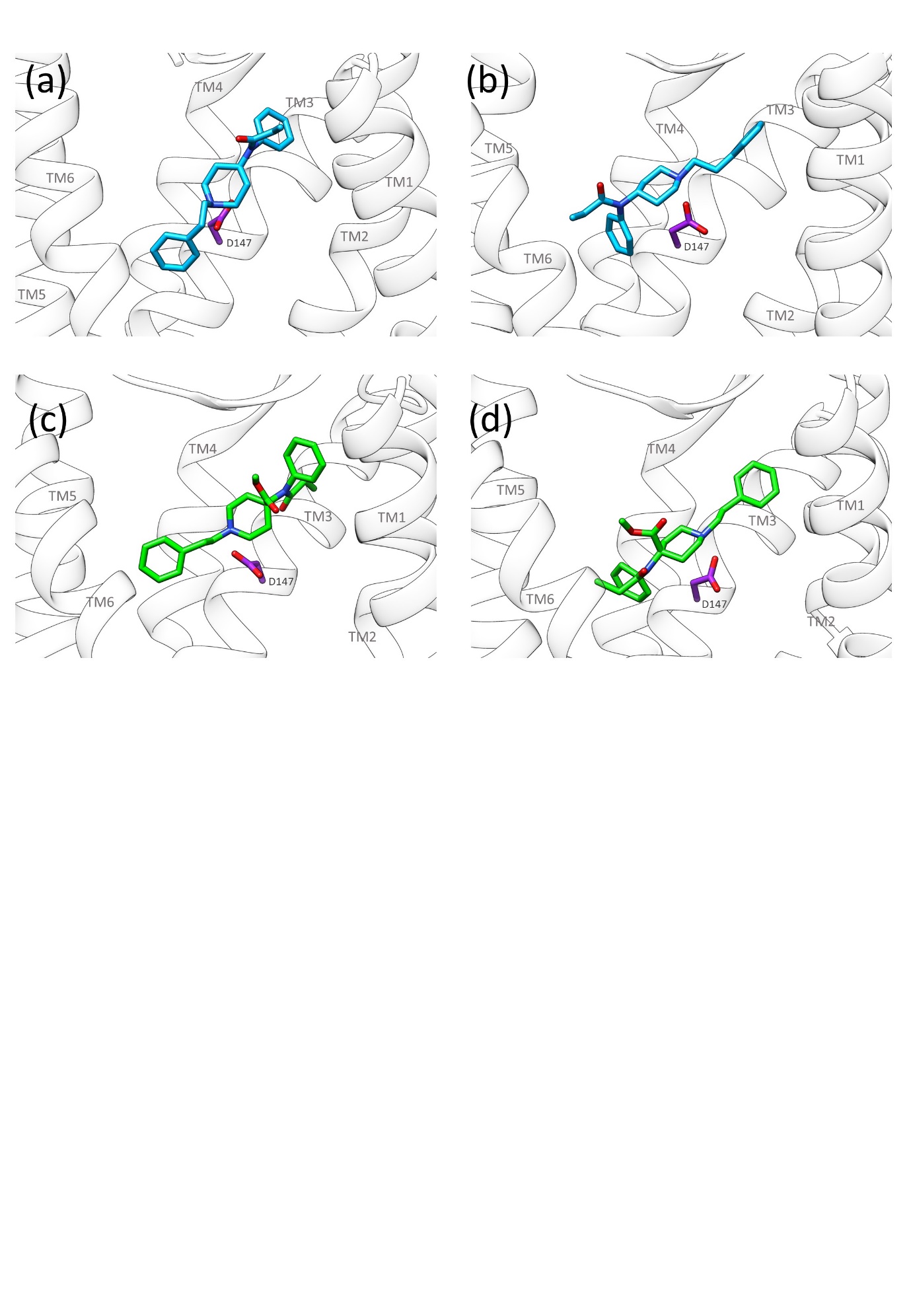
FIGURE S7- Images of fentanyl and carfentanil in the inactive-state µ opioid receptor after 1 µs MD.** Images are shown from the side of the receptor, TMDs have been labelled and TM7 has been removed for clarity. (a) fentanyl pose1, phenethyl towards intracellular side of receptor; (b) fentanyl pose 2, phenethyl towards extracellular side of receptor; (c) carfentanil pose 1, phenethyl towards intracellular side of receptor; (d) carfentanil pose 2, phenethyl towards extracellular side of receptor. Conserved residue ASP147^3.32^ (D147) is shown in purple. Oxygen atoms are shown in red, nitrogen atoms are shown in blue.


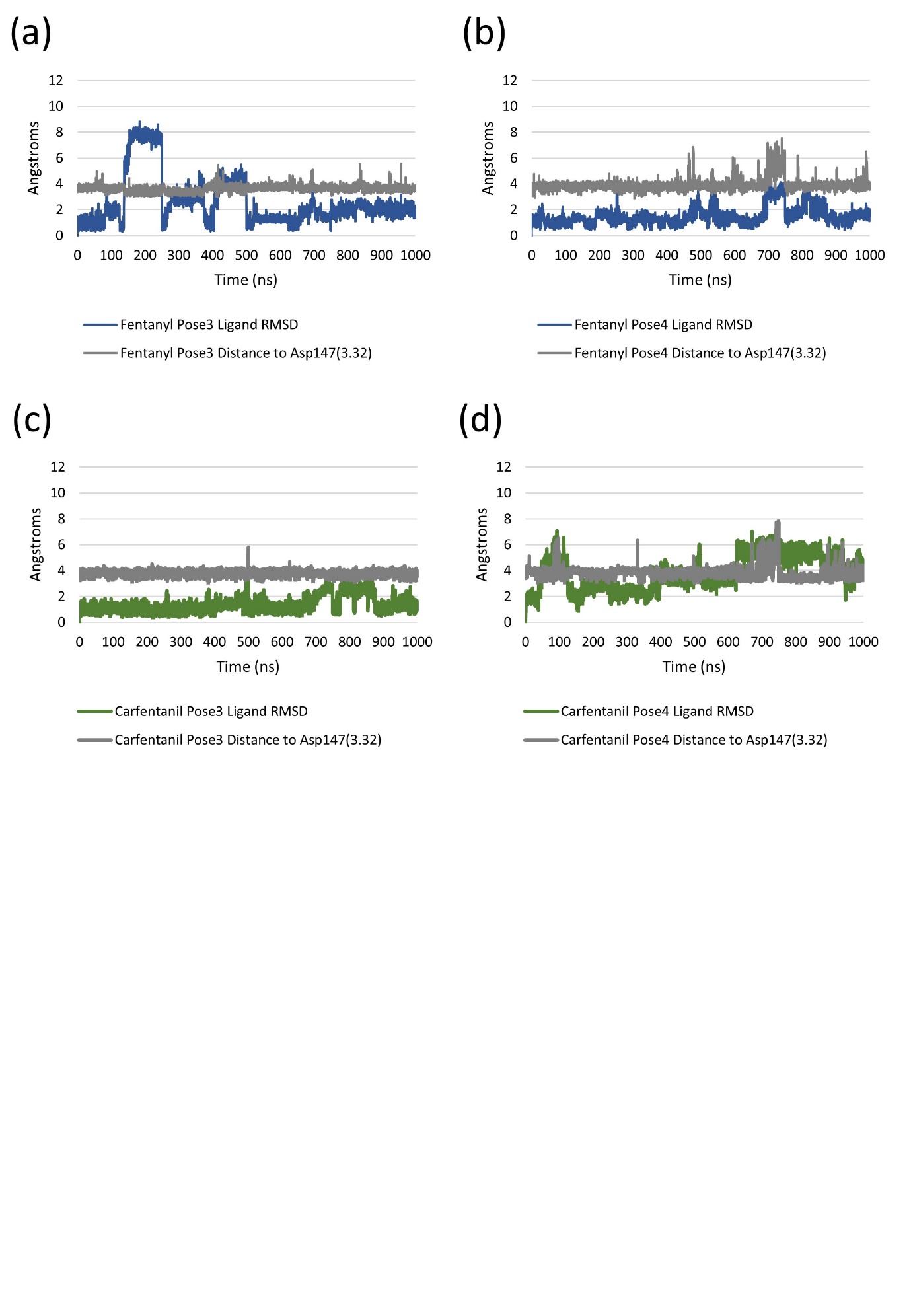
**FIGURE S8- Traces showing the ligand RMSD and proximity to ASP147^3.32^ for 1 µs MDs of fentanyl and carfentanil in the active-state µ opioid receptor structure.** Ligand RMSD is shown in colour for fentanyl pose3 (a, dark blue), fentanyl pose4 (b, dark blue), carfentanil pose3 (c, dark green) and carfentanil pose4 (d, dark green), RMSD is calculated relative to the first frame of the simulation. The distance between the ligand protonated nitrogen and conserved residue ASP147^3.32^ is shown on each plot in grey.


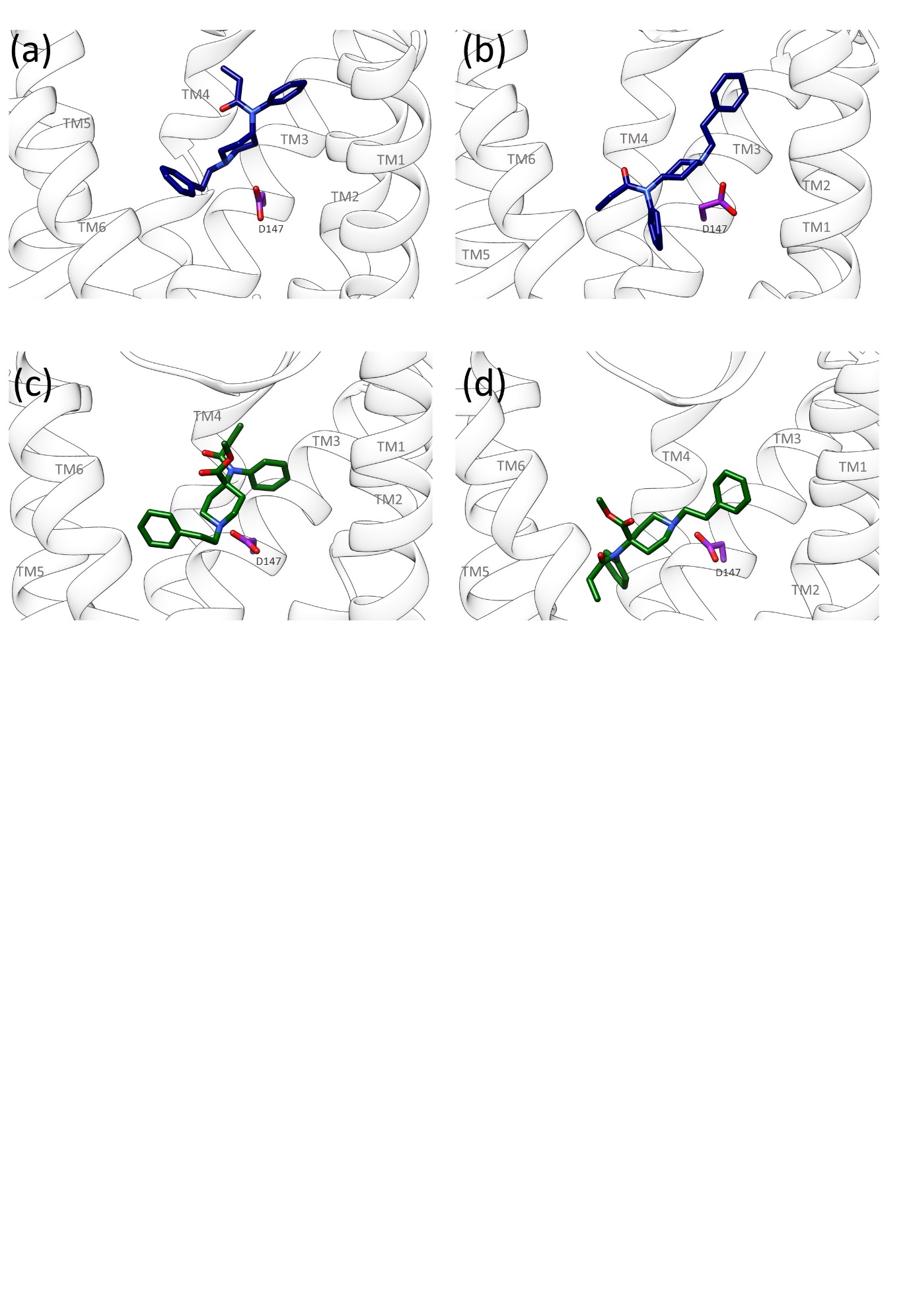


**FIGURE S9-** **Images of fentanyl and carfentanil in the active-state µ opioid receptor after 1 µs MD.** Images are shown from the side of the receptor, TMDs have been labelled and TM7 has been removed for clarity. Images are of the final frame of 1 µs MDs of fentanyl and carfentanil in the active-state receptor (PDB: 5C1M). (a) Fentanyl pose3, phenethyl towards intracellular side of receptor; (b) fentanyl pose 4, phenethyl towards extracellular side of receptor; (c) carfentanil pose 3, phenethyl towards intracellular side of receptor; (d) carfentanil pose 4, phenethyl towards extracellular side of receptor. Conserved residue ASP147^3.32^ (D147) is shown in purple. Oxygen atoms are shown in red, nitrogen atoms are shown in blue.

**
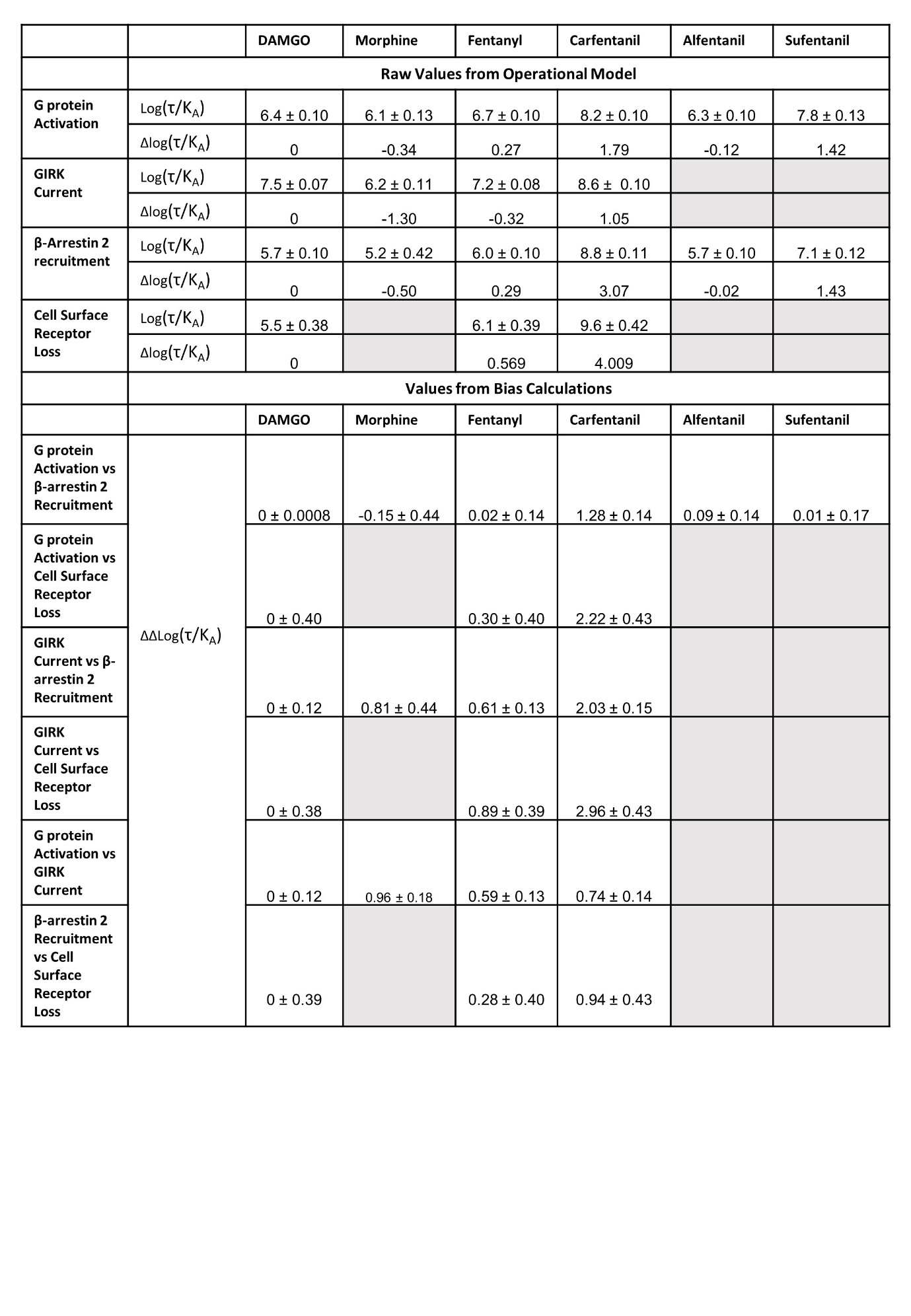
TABLE S1- Table showing the raw values from the bias calculations.** In the table Log(τ/K_A_) values are shown for each agonist in each of the four assays, these values were calculated from fitting the data to the operational model of pharmacological agonism (see section 2.6 in Methods). ΔLog(τ/K_A_) is shown for each agonist, using DAMGO as the reference agonist. ΔΔLog(τ/K_A_) values are also shown for each agonist, for each pathway comparison. Values are means ± SEM. Grey boxes indicate where an agonist was not included in that particular assay.


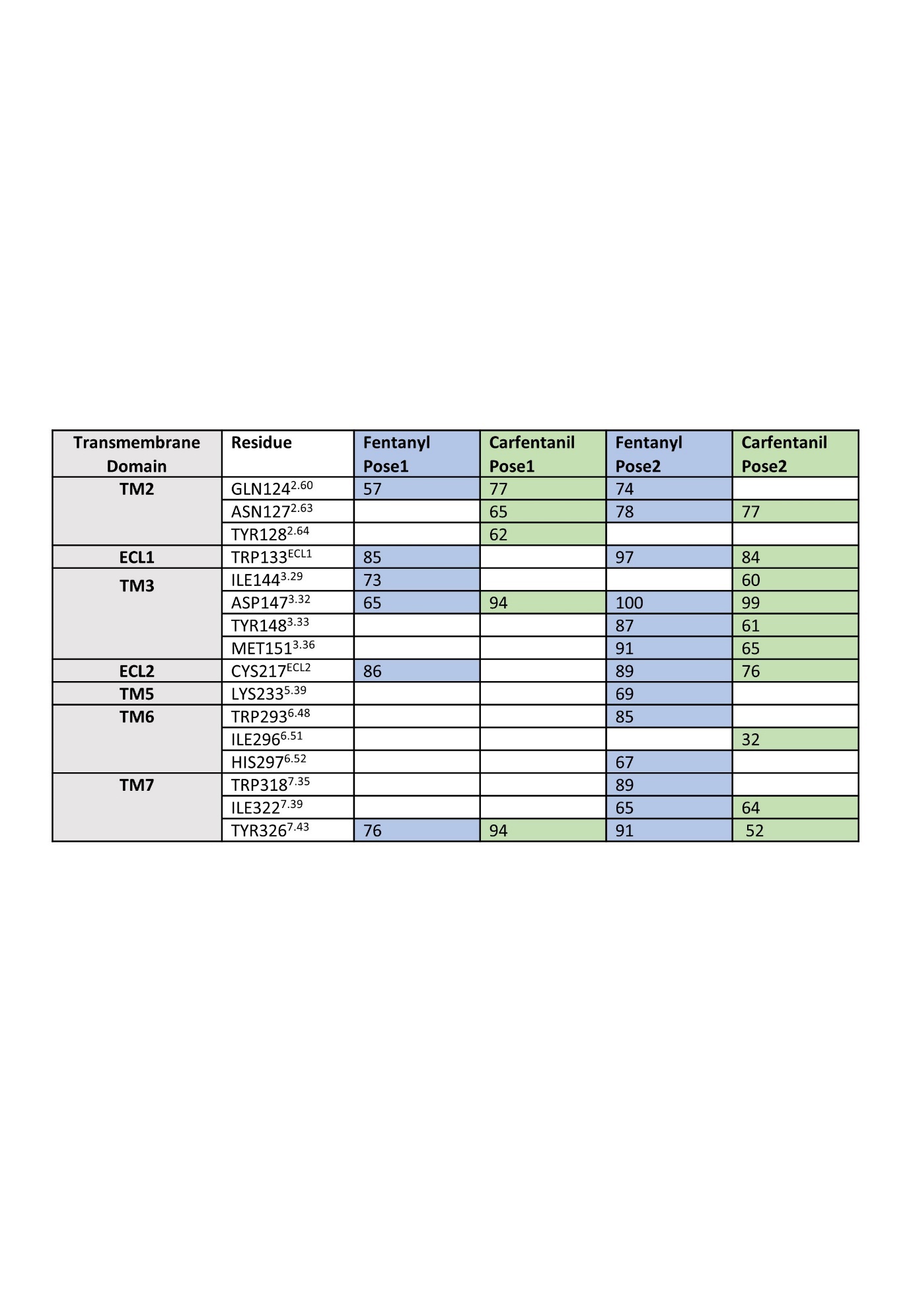


**TABLE S2- Table showing the residues of the μ opioid receptor that were within a 4 Å proximity to fentanyl and carfentanil during the 1 µs MDs in the inactive state µ opioid receptor.** Residues within 4 Å of fentanyl and carfentanil during the 1 µs MDs were calculated for both pose1 and pose2 for the two ligands. The numbers show the % of the MDs that the residue was within 4 Å. The part of the receptor where each residue is found is also indicated. Only residues that were within 4 Å for more than 50% of the MDs time period is shown in order to highlight residues that may be forming stable interactions with the ligands. ILE296^6.51^ has been included for carfentanil Pose2 as, though it is within 4 Å of carfentanil for less than 50% of the simulation, it is still a potentially key interaction with carfentanil’s 4-carbomethoxy group.
